# Deciphering the peripheral immune landscape of Alzheimer’s disease through integrated multi-omics research and cohort validation

**DOI:** 10.3389/fimmu.2026.1648591

**Published:** 2026-04-13

**Authors:** Shi Lv, Jin-yi Kuang, Tian-wei Wang, Li-ping Zhang, Jian Liu, Wen-di Wang, Ming-feng Yang, Qing-bin Ni, Bao-liang Sun, Jing-yi Sun

**Affiliations:** 1 Department of Neurology, Second Affiliated Hospital, Shandong First Medical University & Shandong Academy of Medical Sciences, Taian, Shandong, China; 2 Institute of Brain Science and Brain-inspired Research, Shandong First Medical University & Shandong Academy of Medical Sciences, Jinan, Shandong, China; 3 Qingdao Medical College of Qingdao University, Qingdao, Shandong, China; 4 Department of Neurology, Shandong Second Provincial General Hospital, Jinan, Shandong, China; 5 The Affiliated Taian City Central Hospital of Qingdao University, Taian, Shandong, China; 6 Shandong Provincial Hospital Affiliated to Shandong First Medical University, Jinan, Shandong, China

**Keywords:** Alzheimer’s disease, genome-wide association study, multi-omics Mendelian randomization, peripheral immunity, single-cell transcriptome sequencing, transcriptome-wide association studies

## Abstract

**Objective:**

This study investigated molecular drivers of AD-associated peripheral immune dysregulation to identify pathogenic genes and therapeutic targets for precision diagnosis and intervention.

**Methods:**

A large-scale GWAS meta-analysis (n = 894,710) was performed, followed by two-sample Mendelian randomization (MR) using multi-tissue cis-eQTL data to identify putative causal genes. Immune response differential genes (IRDGs) were defined from AD peripheral blood transcriptomes and the MSigDB database. A three-step summary-data-based MR framework integrating blood cis-eQTLs and cis-mQTLs was applied to prioritize causal genes and epigenetic regulatory elements. Findings were validated through colocalization analysis, PBMC scRNA-seq and blood-tissue TWAS. Multi-dimensional clinical validation was performed in the ADNI cohort encompassing gene expression, CSF biomarkers, cognitive measures, immune cell profiles, survival analysis, and plasma proteomics, with cross-cohort transcriptomic replication in AddNeuroMed.

**Results:**

Two-sample MR identified eight putative AD pathogenic genes. The three-step SMR and colocalization analysis prioritized five candidate causal genes, whose differential expression in immunocytes was confirmed by scRNA-seq and independently replicated. In the ADNI cohort, PTK2B expression was elevated in AD (ANOVA P = 0.0023), inversely correlated with MMSE (r = −0.164, P = 0.017), and predictive of MCI-to-AD conversion (Cox HR = 1.741, P = 0.050), with independent replication in AddNeuroMed (FDR P = 3.56 × 10^−4^). PLEKHA1 and PTK2B expression were strongly associated with peripheral neutrophil and lymphocyte proportions (P < 10^−7^), and PLEKHA1 correlated with CSF total tau (partial r = 0.102, P = 0.036). The prioritized probe cg19863426 at the PLEKHA1 promoter showed progressive hypermethylation across the CN-MCI-AD continuum (F = 3.45, P = 0.032) and was inversely correlated with PLEKHA1 mRNA (r = −0.33, P = 2.68 × 10^−^¹²).

**Conclusion:**

Integrating GWAS, multi-omics Mendelian randomization, single-cell transcriptomics, transcriptome-wide association study, and clinical cohort validation, this study identified peripheral immune causal genes for AD whose blood transcriptomic and epigenetic signatures track with CSF pathology, cognitive decline, and disease progression, supporting their translational potential for early diagnosis and therapeutic development.

## Introduction

Approximately 36 million individuals globally have Alzheimer’s disease (AD), and with the ageing population, this number is projected to increase to over 115 million by 2050 (1). As a neurodegenerative disorder, AD profoundly impairs brain function through substantial loss of synapses and neurons, leading to cognitive and behavioural deficits, along with memory challenges (2). A primary theory about the aetiology of AD posits that dementia and neurodegeneration result from the progressive accumulation of protein aggregates in the brain (3). Previous research indicates that the Aβ cascade hypothesis is the most compelling theoretical model for elucidating AD pathogenesis (4). Therapies aimed at reducing the aberrant aggregation of Aβ or phosphorylated tau (p-Tau) have been evaluated as our comprehension of AD pathogenesis has advanced. Although recent anti-amyloid immunotherapies such as lecanemab have shown modest clinical benefit (5, 6), their limited effect sizes and restricted eligibility criteria underscore the need to explore non-amyloid pathogenic mechanisms (7).

Multiple robust barriers, including the arachnoid mater, cerebrospinal fluid, and blood-brain barrier (BBB), protect the brain from the penetration of material. These barriers protect the brain against external threats, such as toxins and infectious agents, by maintaining a healthy, balanced environment within the central nervous system (CNS). Consequently, the central nervous system has been seen as an “immune-privileged” site. However, as data has accumulated over time demonstrating that acute systemic bacterial or viral infections impact brain function, accumulating evidence has challenged this notion (8). Evidence from preclinical and clinical studies indicates that inflammatory mediators produced throughout the body communicate with the brain through humoral and neural pathways (9). There is increasing recognition of the robust functional connection between the immune system and the central nervous system. Microglia, the predominant immune cells in the neural milieu, can be triggered within the CNS to phagocytose and eliminate β-amyloid (Aβ) while promoting the synthesis of inflammatory cytokines, thereby accelerating neuronal degeneration (10). These data indicate a significant link between the start of AD and immune system malfunction. Additionally, peripheral immunological phenomena interact with the central nervous system in AD. The pathophysiology of AD may involve various immune cell types, including peripheral blood monocytes, neutrophils, macrophages, and T cells, within both the innate and adaptive immune systems (11). The functional status of specific peripheral blood immune cell types in patients with Alzheimer’s disease remains poorly understood. Extensive epidemiological research has shown a correlation between systemic comorbidities and an elevated risk of AD and dementia. Systemic chemicals may influence the biology of AD, neurodegeneration, and cerebral ageing via peripheral pathways that access the brain. Consequently, the genesis and progression of AD involve organs and tissues throughout the body (12–14). Investigating the influence of the peripheral system on the start and course of AD may facilitate the development of novel therapeutic strategies. While genome-wide association studies and blood transcriptomic analyses have implicated several immune-related loci in AD susceptibility (15, 16), whether peripheral expression changes at these loci track with CSF markers of amyloid and tau pathology, cognitive decline, or immune cell composition in the same individuals has not been systematically examined. Closing this gap requires validation in well-characterized clinical cohorts with concurrent gene expression, biomarker, and cognitive data.

The analytical design of this study operates on two distinct levels. At the genome-wide level, a meta-analysis of AD GWAS data from the GWAS Catalogue, FinnGen, and the IEU Open GWAS project was combined with whole-blood cis-expression quantitative trait locus (cis-eQTL) data from GTEx V8 to perform two-sample Mendelian randomization (MR), thereby identifying putative causal genes at AD-associated loci. At the peripheral blood level, a tailored set of immune response differential genes (IRDGs) was constructed by intersecting differentially expressed genes from three AD peripheral blood transcriptome datasets with the GOBP_IMMUNE_RESPONSE gene set retrieved from the Molecular Signatures Database (MSigDB). These IRDGs were then subjected to a three-step summary data-based Mendelian randomization (SMR) framework that integrated blood cis-eQTLs from the eQTLGen consortium (17) and blood cis-DNA methylation QTLs (cis-mQTLs) with the AD GWAS summary statistics, enabling simultaneous inference of transcriptional and epigenetic causality within the peripheral immune compartment. Cell-type resolution was achieved through single-cell RNA sequencing (scRNA-seq) of peripheral blood mononuclear cells (PBMCs) from AD patients and controls, complemented by pseudo-time trajectory analysis to trace expression dynamics across immune cell states. A transcriptome-wide association study (TWAS) using GTEx V8 whole-blood expression weights and the Functional Summary-based Imputation (FUSION) framework provided an independent line of genetic validation. The robustness of the prioritized signals was then assessed through multi-dimensional clinical validation in the Alzheimer’s Disease Neuroimaging Initiative (ADNI) cohort, examining associations between candidate gene expression and CSF amyloid/tau biomarkers, cognitive performance, MCI-to-AD conversion risk, peripheral immune cell proportions, and plasma proteomics. Cross-cohort transcriptomic replication of the gene expression findings was performed in the AddNeuroMed cohort. This layered design progresses from genome-wide causal inference to peripheral immune gene prioritization and, finally, to clinical cohort validation, with each tier providing independent evidence for the translational relevance of the identified targets.

## Materials and methods

### Summary data from GWAS for AD

**Figure 1:**
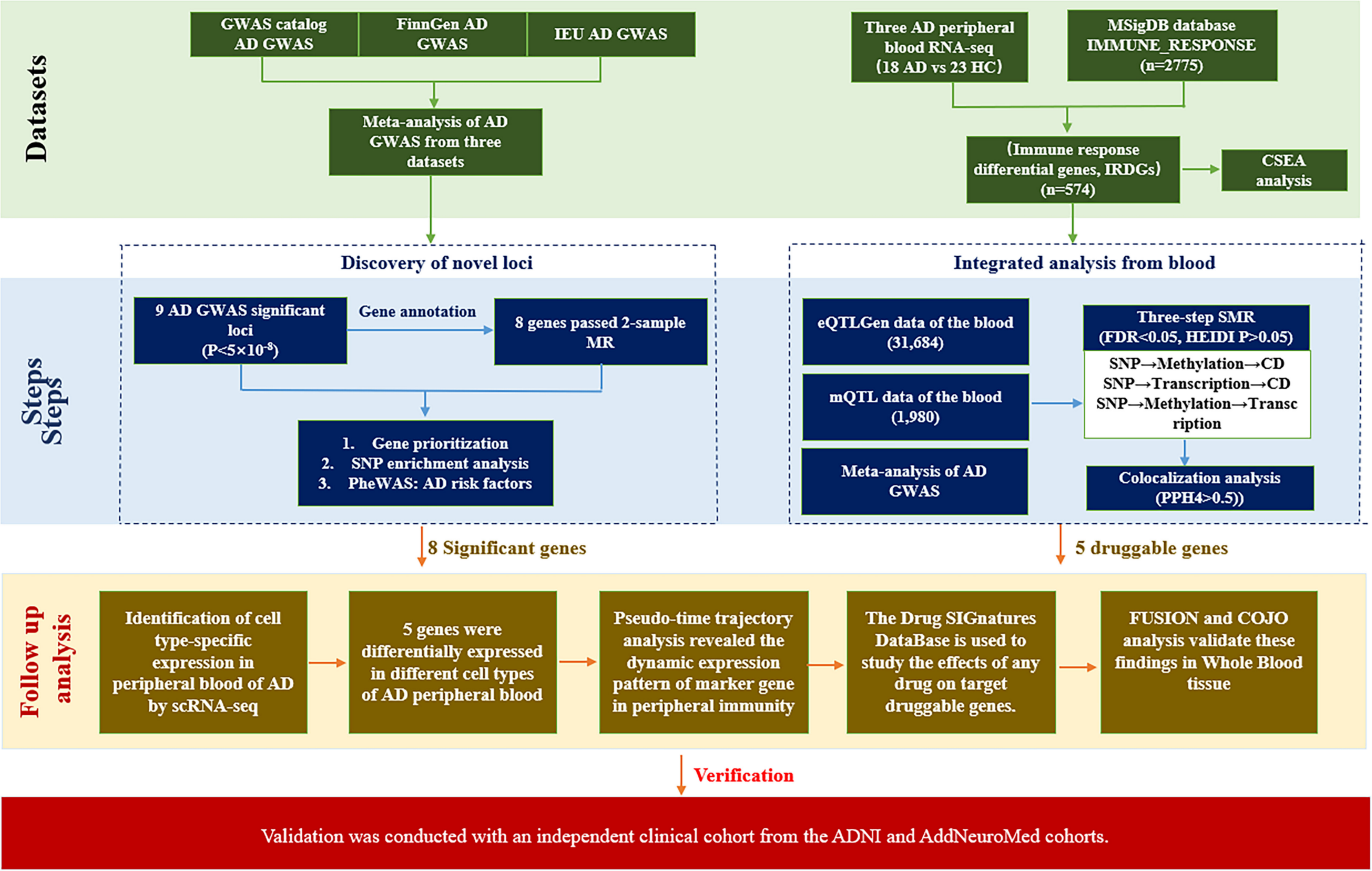
Research flow chart.

This study design is summarised in **Figure 1**. The research population for AD comprises 894,710 participants, of whom 63,926 are from the GWAS catalog (21,982 cases of European ancestry and 41,944 controls), id: GCST007511 (18). 342,499 individuals from FinnGen Round 8 (released on December 1, 2022), id: finngen_R8_G6_ALZHEIMER. The sample size was 342,499 (7,759 cases of European ancestry vs. 334,740 controls). The GWAS for AD diagnosis includes cases reported during discharge or listed as a cause of death for both late-onset and atypical forms. At the same time, unclassified brain degeneration and unspecified dementia were excluded during the quality control phase. 488,285 individuals from the IEU open GWAS project (954 cases of European ancestry and 487,331 controls), Dataset: ieu-b-5067. We conducted a fixed-effects inverse-variance-weighted meta-analysis of AD using the GWAS catalog (n = 63,926), the FinnGen database (n = 342,499), and the IEU database (n = 488,285), totalling 894,710 individuals in the GWAS, using METAL software (version released on 2020-05-05) (19). We excluded variants with MAF < 0.5%, yielding 698,416 associations. A MAF threshold of 0.5% was applied to retain low-frequency variants with potential biological relevance, given the large combined sample size, which provided adequate power to detect them. To assess the potential impact of sample overlap across the three GWAS sources, we performed a sensitivity analysis using the FinnGen R11 cohort as an independent replication dataset. The FinnGen R11 release represents a substantial update over the Round 8 data included in our meta-analysis, with a larger sample size and refined phenotype definitions. The FinnGen biobank recruits participants exclusively through the Finnish healthcare system and does not share samples with the GWAS Catalog or IEU Open GWAS project. Genome-wide significant loci identified in the meta-analysis were queried in FinnGen R11, and effect alleles were harmonized to a common orientation before assessing directional consistency and nominal significance.

We got three RNA-seq datasets (GSE97760, GSE168813, and GSE203408) from the GEO database (AD patient, PBMCs untreated, and Control, PBMCs untreated). These datasets provided peripheral blood expression profiles of AD exclusively derived from human samples. Transcriptomic data from all samples were batch-corrected and visualized using principal component analysis (PCA). The limma R package was employed to identify peripheral blood differentially expressed genes (DEGs) between AD patients and healthy controls, with thresholds set at (log_2_ FC) > 1 and adjusted P < 0.05. The immune response gene set ‘GOBP_IMMUNE_RESPONSE’ (ARCHIVED C5_GO, Homo sapiens) was retrieved from the MSigDB database. Intersection genes between ‘GOBP_IMMUNE_RESPONSE’ and DEGs were defined as immune response differential genes (IRDGs) in AD peripheral blood. The Cell Type-Specific Enrichment Analysis Database (CSEA-DB, https://bioinfo.uth.edu/CSEADB/) was used to assess whether IRDGs were specific to any immune cell type.

We annotated the findings using FUMA (20) with default settings. We identified specific variants with R² < 0.6 using standard FUMA parameters and uncovered associations with index variants exceeding 500 KB in previously reported index variants across the FinnGen, IEU, and GWAS catalog datasets. The priority (P < 5 × 10^-8^) locus for the GWAS-essential genes was identified using the gene most similar to the index variant and the principal gene at each locus, as determined by the Polygenic Priority Score (PoPS). Subsequently, we identified genes with significant enrichment for AD GWAS variant SNPs in GTEx V8 blood and brain tissues.

### Correlation of AD GWAS variants with characteristics of AD risk factors

We identified genetic associations for variants exceeding the AD GWAS threshold (P < 5 × 10^-8^) in GWAS of seven AD risk factors. AD risk factor data were sourced from the GWAS research of European descent: smoking, insomnia, hypertension, diabetes, depression, anxiety, and alcohol (Finngen Round 8, released on 1 December 2022). We used a P-value < 0.05 for correlation analysis to assess whether the directionality of relationships with AD risk factors matched the results for AD. Specifically, we expected a positive correlation with harmful risk factors for variants that were linked to a higher risk of AD.

### Summary statistics of eQTL

Genetic variants that influence gene expression levels at specific loci are termed expression quantitative trait loci (eQTLs). Characterizing eQTLs clarifies the regulatory architecture linking genotype to transcription and, by extension, to disease phenotype (21). For the genome-wide MR screening of putative causal genes at AD-associated loci, whole blood cis-eQTL summary statistics were obtained from the GTEx project V8 (n = 670; https://gtexportal.org/home/) (22). Independent cis-eQTLs satisfying FDR < 0.05 and located within 1 Mb of the transcription start site were retained as genetic instruments, with a significance threshold of P < 1 × 10^−5^ applied to the MR analysis. For the subsequent three-step SMR analysis focused on peripheral blood IRDGs, blood cis-eQTL data were sourced from the eQTLGen consortium (n = 31,684) (17), which was selected over GTEx V8 blood owing to its substantially larger sample size and correspondingly greater statistical power to detect blood-specific regulatory signals. For the TWAS validation, pre-computed whole-blood gene expression weights from GTEx V8 were used, as required by the FUSION analytical framework. Blood cis-mQTL summary statistics were derived from a meta-analysis of the Lothian Birth Cohorts and the Brisbane Systems Genetics Study (23). All analyses were restricted to cis-acting variants within 1 Mb of the target gene’s transcription start or end sites, ensuring a focused investigation of proximal regulatory mechanisms.

To prioritize candidate causal genes at each genome-wide significant locus, we applied the Polygenic Priority Score (PoPS) method (24), which integrates GWAS summary statistics with gene expression profiles, biological pathway annotations, and predicted protein-protein interaction networks. A 500-kb window around each index variant was used to map GWAS loci to Ensembl genes, and the gene with the highest PoPS score within each locus was selected as the prioritized candidate.

### Three-step SMR and colocalization analysis

The SMR test assesses the association between an exposure and an outcome using a single genetic variant as an instrumental variable. The detailed method has been described in the study by Zhu et al. (25). In the blood tissue analysis, the SMR multi-instrument was used to make causal inferences for IRDGs, and the 1000 Genomes European reference was used to determine linkage disequilibrium (LD). A three-step SMR analysis was performed: (1) IRDGs were considered as the exposure, and AD was the outcome;(2) Blood DNAms was the exposure, and AD was the outcome;(3) Blood DNAms was the exposure, and IRDGs were the outcome. Step 3 only included the significant signals from Steps 1 and 2. The final candidate signals were defined as follows:(1) Passed the false discovery rate (FDR) < 0.05 in all three steps of the SMR analysis;(2) Showed genome-wide significance (P < 1 × 10^−5^) in all eQTL, mQTL, and GWAS data;(3) Exhibited heterogeneity in the dependent instrument (HEIDI) test results, with P > 0.05.Colocalization analysis aimed to identify overlapping variants that may contribute to different traits. The R package “coloc” was used for colocalization analysis (26), with PP.H4 > 0.5 as the threshold for shared genetic effects between two traits (27).

### Expression analysis at the single-cell level

To further assess cell-type-specific expression of candidate genes with putative causal effects on AD, we analysed scRNA-seq data from PBMCs of AD patients and healthy controls (GSE181279; 3 AD cases and 2 controls) (28). Raw scRNA-seq data were preprocessed using the Seurat R package (29), and genes detected in <3 Cells and cells with <50 unique molecular identifiers (UMIs) were excluded, and transcripts per million (TPM) were normalized and scaled via the NormalizeData and ScaleData functions. Manually annotated cell types were analysed for differential expression of AD-associated protein-coding genes (including 10 known AD risk loci) using Wilcoxon rank-sum tests (|log2FC| > 1, adjusted P < 0.05). For pseudo-time trajectory analysis, Seurat-processed data were imported into Monocle 2 via importCDS(object, import_all = FALSE), normalized [estimateSizeFactors()], and analysed for gene dispersion [estimateDispersions()]. Genes with cluster-specific differential expression [q < 0.01, differentialGeneTest(fullModelFormulaStr = “~clusters”)] were selected as ordering genes. Dimensionality reduction [reduceDimension(max_components = 2, reduction_method = “DDRTree”)] and pseudotime inference [orderCells()] revealed transcriptional state trajectories. Key genes (e.g., PLEKHA1 and PTK2B, linked to AD-associated NK cell dysfunction) were visualized along pseudotime to track expression dynamics. To validate the discovery scRNA-seq findings and address the limited sample size of the initial dataset, we incorporated an independent, large-scale PBMC scRNA-seq dataset (GSE226602) from Ramakrishnan et al. (9), comprising 50 age-, sex-, and APOE-matched individuals (28 AD, 22 healthy controls). Raw count matrices were downloaded from GEO and processed using the Seurat pipeline with quality control filters (200 < nFeature < 6,000; mitochondrial fraction < 15%). After log-normalisation, the top 2,000 variable features were selected, and cells were clustered at a resolution of 0.5 using PCA (30 components) and UMAP embedding. Cell types were annotated into 19 populations based on canonical marker expression. To validate candidate gene cell-type enrichment, expression profiles of all 15 candidate genes were examined across annotated cell types using DotPlot visualization. Differential expression between AD and HC within each cell type was assessed using Wilcoxon rank-sum tests (min.pct = 0.01, logfc.threshold = 0.5). For NK cell sub-analysis, NK cells were extracted and re-clustered (resolution 0.4, 15 PCs), yielding subclusters annotated into nine functional subtypes based on top differentially expressed markers. Compositional differences in NK subtypes between AD and HC were evaluated using Fisher’s exact test with Benjamini-Hochberg correction. It should be noted that both the discovery and validation datasets were derived from PBMC preparations using Ficoll density gradient centrifugation, which physically excludes granulocytes, including neutrophils, due to their higher buoyant density. Finally, the Drug Signatures Database (https://dsigdb.tanlab.org/) was queried to identify druggable targets.

### Single-tissue transcriptome-wide association study analysis

We used the FUSION framework (http://gusevlab.org/projects/fusion/) to conduct a TWAS by integrating AD GWAS summary statistics with precomputed gene expression weights from GTEx V8 blood tissue (30). Candidate genes were retained if they reached FDR < 0.05 in the cross-tissue TWAS and FDR < 0.05 in at least one tissue in the single-tissue analysis. To distinguish independent genetic signals from those driven by linkage disequilibrium, we performed conditional and joint (COJO) analysis using the FUSION post-processing module (31).

### Independent clinical cohort validation

Peripheral blood gene expression data for the validation analysis were drawn from the ADNI database. The ADNI database provided Illumina HumanHT-12 v3.0 BeadChip profiles (49,386 probes, 745 samples), longitudinal clinical diagnoses, demographic and APOE genotyping data, cerebrospinal fluid (CSF) biomarker measurements (Roche Elecsys immunoassay for amyloid-β42 [Aβ42], total tau, and phosphorylated tau), Mini-Mental State Examination (MMSE) scores, complete blood count differentials, and Rules-Based Medicine multiplex plasma proteomic assays. For multiple probe sets mapping to the same gene symbol, expression values were averaged to yield a single value per gene per sample. After filtering and removing records with missing group assignment, 550 participants remained (200 CN, 284 MCI, 66 AD). Continuous variables were screened for outliers at ±3 standard deviations, and then Box-Cox transformed to approximate normality, with a constant shift applied when non-positive values were present. Blood count records from the ADNI LABDATA file, in which missing values were encoded as −1, were recoded and mapped from internal codes to standard haematology variables, then merged by subject identifier, retaining the earliest available record per participant (543/550 matched, 98.7%). Plasma proteomic data were linked via subject-level identifier mapping, yielding 144 matched participants. Blood DNA methylation data for the two CpG sites were derived from ADNI Illumina 450K array raw IDAT files following standard preprocessing protocols, including background correction, normalisation, and probe filtering. The resulting methylation β-values were matched to the analytic dataset by subject identifier. To confirm transcriptomic reproducibility across independent populations, we used the AddNeuroMed study, a European multi-centre cohort with blood expression data from two GEO series (GSE63060 and GSE63061, Illumina HumanHT-12 v3/v4 BeadChip, 717 participants: 240 CN, 192 MCI, 284 AD) and matched clinical annotations (15). The ADNI gene expression validation was structured across six analytic dimensions. Linear regression tested covariate-adjusted group differences in transformed expression (CN as the reference), controlling for age, sex, and APOE ϵ4 carrier status, using Type III sums of squares and Bonferroni-corrected pairwise contrasts based on estimated marginal means. One-way analysis of variance assessed overall group effects, and a linear trend was evaluated by regressing expression on an ordinal diagnostic variable. Pearson, Spearman, and partial correlations, adjusting for age, sex, and APOE ϵ4, quantified associations between transformed gene expression and CSF Aβ42, total tau, phosphorylated tau, their ratios, and MMSE scores. Among 214 baseline MCI participants with gene expression data (64 converters within 5 years), Cox proportional hazards models estimated hazard ratios for MCI-to-AD conversion, sequentially adjusting for age, sex, and APOE ϵ4 status. The log-rank test was used to compare Kaplan-Meier curves stratified by median expression. Standardized regression assessed associations between gene expression and neutrophil and lymphocyte percentages, and tested whether these immune markers were in turn associated with CSF biomarkers and cognitive scores. For plasma proteomics, each of 146 analytes was regressed on gene expression (adjusted for age and sex) with false discovery rate (FDR) correction across all tests per gene, and proteins that reached nominal significance (P < 0.05) were further tested for group differences using Type III analysis of covariance. Subgroup analyses stratified by sex (male versus female) and age (<75 versus ≥75 years) included formal Group × Sex and Group × Age interaction terms to evaluate sex-specific or age-specific effects. The methylation analysis followed the same statistical framework, applying identical covariate adjustments, subgroup stratifications, interaction evaluations, and sensitivity checks. Sensitivity analyses across all dimensions included APOE ϵ4 stratification, interquartile-range-based outlier exclusion, and Bonferroni and FDR corrections for primary tests. For the AddNeuroMed replication, analysis-of-covariance models regressed probe-level expression on diagnostic group, adjusting for age and sex, and correcting for multiple testing across genes. Statistical significance was defined as two-sided P < 0.05 unless otherwise noted.

## Results

### A genome-wide meta-analysis reveals nine significant loci associated with AD

**Figure 2:**
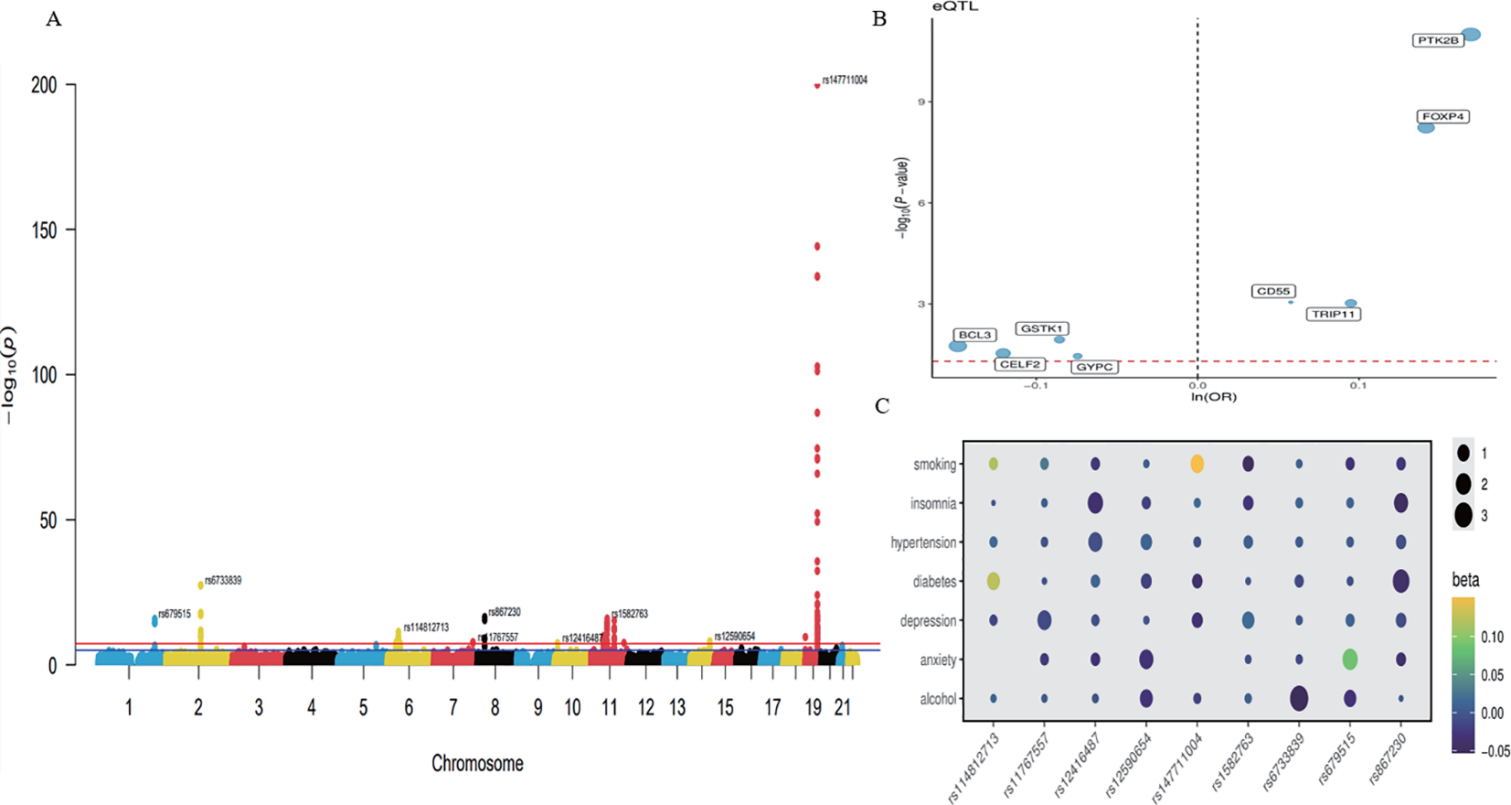
**(A)** Manhattan plot showing the correlation between SNPs and AD. Manhattan plots illustrate the -log10(p-value) relationships for each SNP from GWAS meta-analyses, with chromosomal position shown on the y-axis. The red dashed lines represent the criteria for genome-wide significance (P < 5×10^−8^). **(B)** MR analysis of eQTL and AD summary data of 8 significant genes. The right side indicates a positive association with AD risk, whereas the left side indicates a negative association. The red dashed lines represent the criteria (P < 0.05). **(C)** The genetic association between 9 AD loci and AD risk factors.

We conducted a meta-analysis of GWAS data on AD from the GWAS Catalog, the FinnGen database, and the IEU Open GWAS project. Following quality control, we identified 11,478,345 genetic variants associated with AD. We identified 9 genome-wide significant AD-associated loci, of which 4 were novel, and 5 had been previously reported (**Table 1**; **Figure 2A**). The quantile-quantile (Q-Q) plot of the meta-analysis is presented in **Supplementary Figure S1**. Whole blood cis-eQTL data from GTEx V8 were extracted for both the nearest genes and all genes prioritized by PoPS at each of the nine genome-wide significant loci (**Supplementary Tables S1**, **S2**). Independent cis-eQTLs satisfying FDR < 0.05 were retained as genetic instruments. Two-sample MR integrating these instruments with the AD meta-GWAS summary statistics identified eight genes with evidence of causal association (**Figure 2B**). Four genes exhibited a positive association with AD risk, namely PTK2B (OR = 1.18, FDR = 7.99 × 10^−^¹¹), FOXP4 (OR = 1.07, FDR = 2.32 × 10^−8^), TRIP11 (OR = 1.10, FDR = 1.89 × 10^−^³), and CD55 (OR = 1.06, FDR = 1.89 × 10^−^³), while four genes were inversely associated, namely BCL3 (OR = 0.71, FDR = 2.35 × 10^−^²), GSTK1 (OR = 0.92, FDR = 1.82 × 10^−^²), CELF2 (OR = 0.89, FDR = 3.33 × 10^−^²), and GYPC (OR = 0.93, FDR = 3.52 × 10^−^²). All instruments passed Steiger filtering, confirming that the selected variants explained a greater proportion of variance in gene expression than in AD liability and thereby reducing the likelihood of reverse causation. The mean F-statistic across all instruments exceeded 10, indicating that weak-instrument bias was unlikely to materially affect the estimates (**Supplementary Table S3**; **Supplementary Figures S2**–**S9**). SNP enrichment analysis identified four pathways. For the Gene Ontology function annotation of genes, Biological Process (BP) enriched 459 phrases, Cellular Component (CC) enriched 53 terms, and Molecular Function (MF) enriched 54 terms (**Supplementary Figure S10**). **Figure 2C** shows a correlation between several AD forms and at least one risk factor for the condition. We linked four variations to two AD risk factors: rs12416487/CELF2 (insomnia,hypertension), rs12590654/TRIP11/ATXN3 (anxiety, alcohol), rs679515/CD55(anxiety, alcohol), and rs867230/PTK2B (insomnia, diabetes). All variations and relationships with AD risk factors showed a tendency toward AD (**Supplementary Table S4**). To evaluate the robustness of these loci against potential overlap-induced bias, all nine genome-wide significant variants were queried in the independent FinnGen R11 cohort. After allele harmonization, seven of nine loci reached nominal significance (P < 0.05), and five showed directionally consistent effects with the meta-analysis (**Supplementary Table S5**). The loci harbouring PTK2B (rs867230, P = 1.19 × 10^−5^), CD55 (rs679515, P = 1.33 × 10^−6^), CELF2 (rs12416487, P = 6.51 × 10^−5^), and MS4A1 (rs1582763, P = 1.52 × 10^−8^) all demonstrated directionally concordant and statistically significant associations, confirming the robustness of these signals. Two loci (rs147711004 near BCL3 and rs6733839 near BIN1) showed directional discordance despite strong statistical significance in both datasets. The FOXP4 locus (rs114812713) showed directional consistency but did not reach nominal significance (P = 0.85), consistent with the low effect allele frequency of this variant in the Finnish population (EAF = 0.01).

**Table 1:** Genome-wide significant AD-associated loci and prioritized genes from GWAS meta-analysis.

rsID	Chr	Pos	Nearest Gene	PoPs gene	Pops_score	Effect_allele	Other_allele	Beta	Se	P
Novel variants										
rs114812713	6	41066261	FOXP4	FOXP4	0.349836	C	G	1.9560	0.1322	4.47E-12
rs12416487	10	11679058	CELF2	CELF2	0.644368	A	T	-1.4796	0.1512	3.42E-08
rs12590654	14	92472511	TRIP11	ATXN3	0.047182	A	G	1.7219	0.2992	8.73E-09
rs147711004	19	44834661	BCL3	BCL3	0.993335	A	G	-1.9982	0.2789	1.00E-190
rs867230	8	27610986	PTK2B	PTK2B	0.395930	A	C	3.9869	0.4730	3.49E-17
Previously reported variants										
rs11767557	7	143412046	GSTK1	GSTK1	0.302752	T	C	-1.2655	0.2237	1.56E- 08
rs1582763	11	60254475	MS4A1	MS4A1	1.153671	A	G	-2.1264	0.25666	1.19E-16
rs6733839	2	127135234	BIN1	BIN1	0.493517	T	C	-1.5318	0.1393	4.02E-28
rs679515	1	207577223	CD55	CD55	0.598733	T	C	1.8497	0.2241	1.56E-16

### Screening key IRDGs

**Figure 3:**
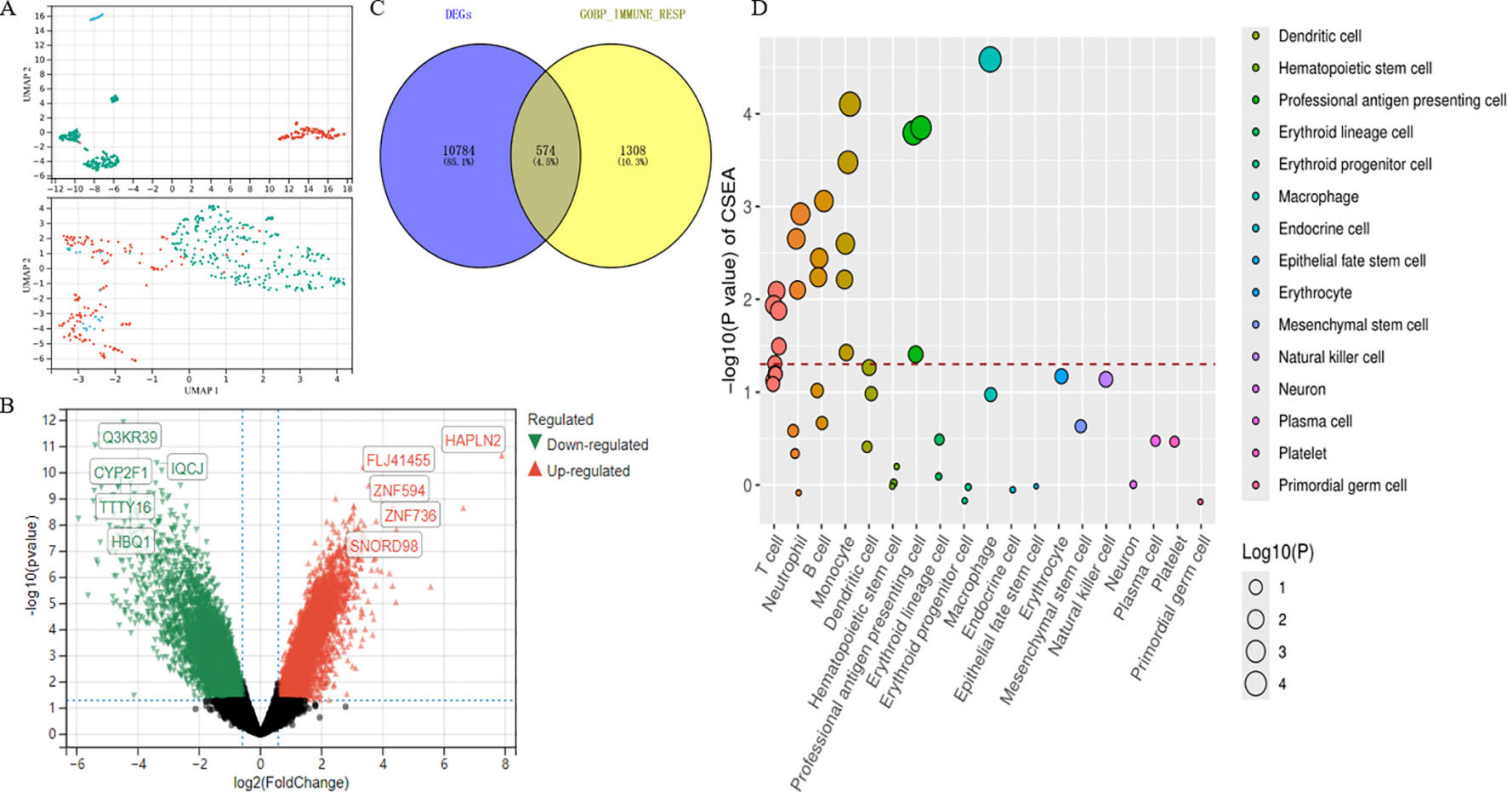
**(A)** PCA plots of the bulk transcriptome sequencing dataset before and after the removal of batch effects. **(B)**The volcano plots illustrate the differentially expressed genes in AD and control peripheral blood samples. Black dots denote genes with non-significant expression. **(C)** Venn diagram of DEGs and immune response genes. **(D)** A cell-type-specific enrichment analysis database was used to assess the specificity of IRDGs across brain tissue types and peripheral blood cells. The x-axis shows the various types of cells found in brain tissue and blood. It shows 23 broad categorization annotations ordered from most important to least important. The dashed line represents the effective threshold for an FDR < 0.05.

We obtained three RNA-seqs from the GEO database (GSE97760, GSE168813, GSE203408). PCA revealed heterogeneity in both AD and healthy control peripheral blood samples before and after debatching (**Figure 3A**). Bulk RNA-Seq analysis identified 11,358 DEGs, comprising 4,417 upregulated and 6,941 downregulated DEGs (**Supplementary Table S6**). The volcano plot illustrates the distribution of DEGs (**Figure 3B**). 2,775 linked to immune response were retrieved from the MSigDB database (**Supplementary Table S7**). A Venn diagram revealed 574 IRDGs, with the top five upregulated genes being KIR2DL4, CAV1, IFNG, IL18R1, and POU2F2, while the downregulated genes included CTLA4, RNF19B, IL6ST, MAD2L2, and MAPK8 (**Figure 3C**). We conducted CSEA of IRDGs across 126 general cell types from 111 tissues. Focusing on peripheral blood cells, we identified seven tissues and 15 general cell types after adjusting for multiple comparisons using the BH method (FDR < 0.05). The IRDGs were significantly enriched for monocyte-macrophage markers (**Supplementary Table S8**), indicating their crucial role in maintaining homeostasis in the peripheral environment of AD (**Figure 3D**).

### Integration of blood-based GWAS and IRDGs-associated cis-eQTL/mQTL data

Given the differential expression of multiple genes in the peripheral blood of AD patients, we hypothesize that altered gene expression may underlie the disease’s causal mechanisms. Furthermore, DNAm in promoter or enhancer regions is known to modulate the regulation of disease-associated target genes. We aimed to identify candidate causal genes in the peripheral blood of AD patients and explore their potential epigenetic regulatory mechanisms. A three-step SMR approach was employed, retaining only significant signals that passed sensitivity analyses across all three steps as putative causal genes. Here, we integrated cis-eQTLs and cis-mQTLs for 574 IRDGs in AD peripheral blood with AD GWAS summary statistics to prioritize genes with concordant evidence across genetic, transcriptional, and epigenetic layers.

**Figure 4:**
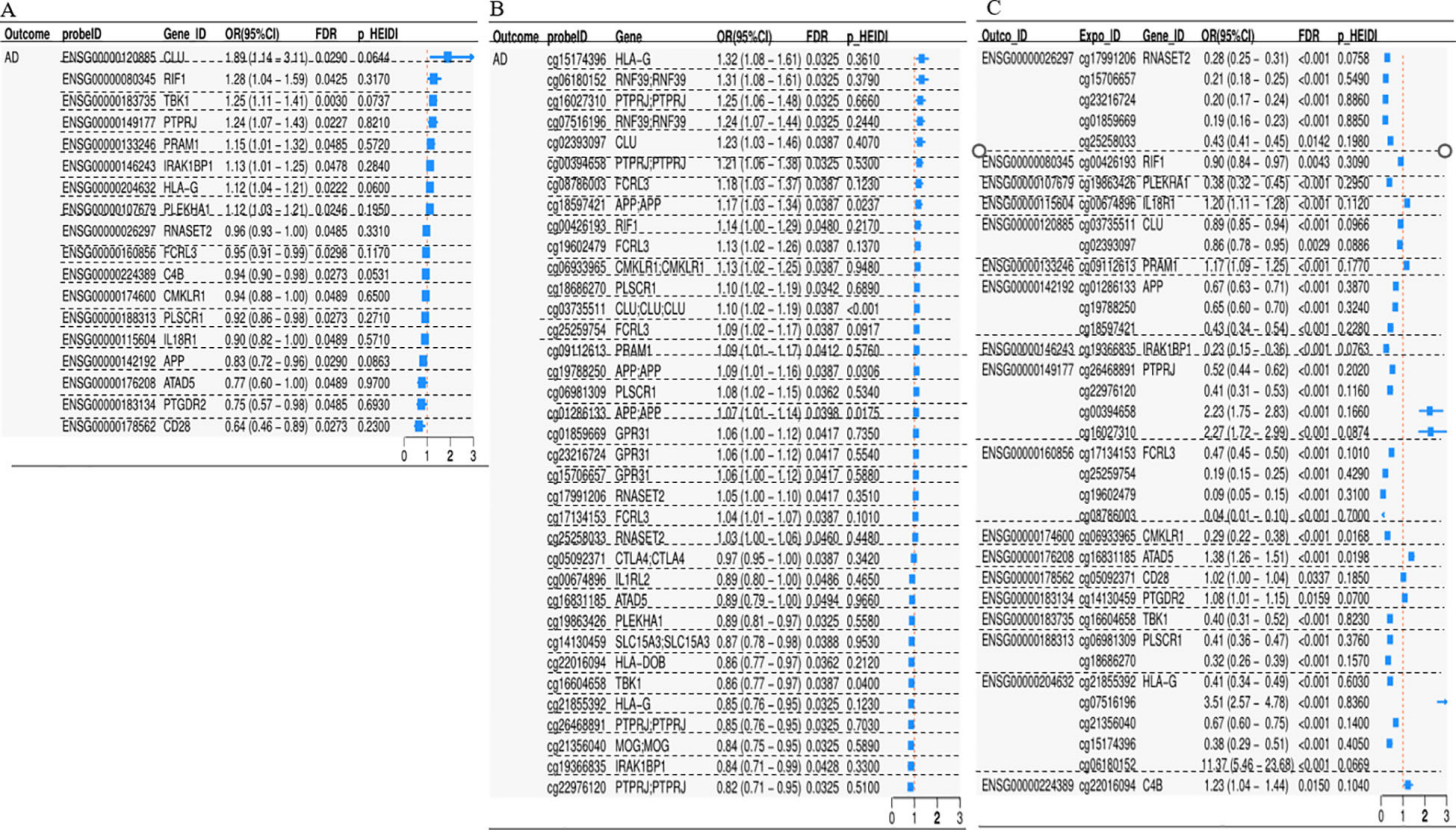
Forest plot illustrating the outcomes of merging eQTL/mQTL data pertinent to GWAS and IRDGs in blood. **(A–C)** corresponds to stages 1–3 of the formulated three-step SMR.

Integration of blood cis-eQTL data with AD GWAS summary statistics identified 45 IRDGs (**Supplementary Table S9**, Step 1). Subsequent integration of the same AD GWAS data with cis-mQTL summary statistics revealed 159 DNAm probes (**Supplementary Table S10**, Step 2). Further cross-omics integration prioritized 36 DNAm probes (**Supplementary Table S11**, Step 3), regulating 18 putative causal genes (SMR FDR < 0.05, HEIDI P > 0.05) through concordant cis-eQTL and cis-mQTL signals (**Figures 4A–C**). Colocalization analysis identified shared genetic variants between gene expression (eQTL) and AD risk, with five genes—PLEKHA1 (PP.H4 = 0.897), TBK1 (PP.H4 = 0.896), APP (PP.H4 = 0.800), PTGDR2 (PP.H4 = 0.693), and CD28 (PP.H4 = 0.536)—exhibiting robust genetic causality (**Supplementary Table S12**; **Supplementary Figure S11**).

### Proposed blood methylation modulation of gene expression in AD’s causative genes

**Figure 5:**
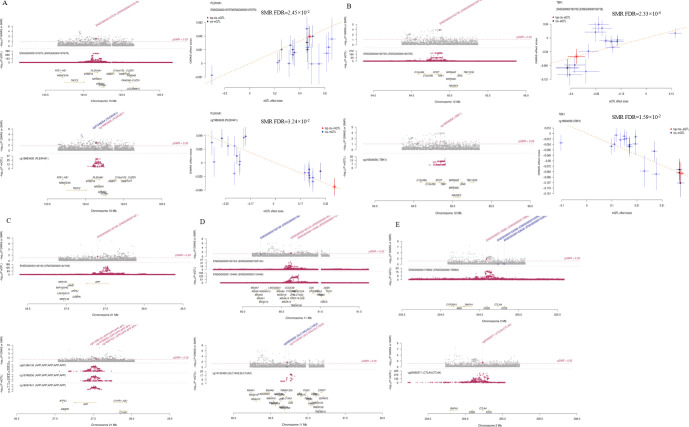
SMR locus zoom-in view of putative risk genes in blood. The locus-wide plot displays concordant genetic effects from AD GWAS, cis-mQTL, and cis-eQTL data near the gene (minimum *P* < 1 × 10^−5^). **(A–E)** illustrate regulatory mechanisms for PLEKHA1, TBK1, APP, PTGDR2, and CD28, respectively.

Our three-step SMR analysis prioritized PLEKHA1, where the DNAm probe cg19863426, located within the promoter region ~4.629 kb upstream of the TSS, exhibited inverse associations with PLEKHA1 expression (OR = 0.38, [95% CI: 0.32–0.45], FDR = 1.53 × 10^−27^) and AD risk (OR = 0.89, [95% CI: 0.81–0.97], FDR = 3.25 × 10^−^²). Conversely, PLEKHA1 expression was positively associated with AD (OR = 1.12, [95% CI: 1.03–1.21], FDR = 2.46 × 10^−^²) (**Figure 5A**). For TBK1, the intragenic DNAm probe cg16604658 (disrupting transcription factor binding) showed inverse correlations with TBK1 expression (OR = 0.40, [95% CI: 0.31–0.52], FDR = 1.15 × 10^−^¹²) and AD risk (OR = 0.86, [95% CI: 0.77–0.97], FDR = 3.87 × 10^−^²). The positive association between TBK1 expression and AD (OR = 1.25, [95% CI: 1.11–1.41], FDR = 3.00 × 10^−^³) supports a model in which hypermethylation suppresses TBK1 transcription, thereby mitigating AD pathogenesis (**Figure 5B**). Three DNAm probes (cg01286133, cg19788250, cg18597421) near the 3′ terminus of APP on chromosome 21 were linked to increased AD risk (OR = 0.83, [95% CI: 0.72–0.96], FDR = 2.90 × 10^−^²), likely through transcriptional termination interference and negative regulation of APP expression (**Figure 5C**). The upstream DNAm probe cg14130459 of PTGDR2 positively correlated with its expression (OR = 1.08, [95% CI: 1.01–1.15], FDR = 1.59 × 10^−^²), while both the probe (OR = 0.87, [95% CI: 0.78–0.98], FDR = 3.88 × 10^−^²) and PTGDR2 expression (OR = 0.75, [95% CI: 0.57–0.98], FDR = 4.85 × 10^−^²) inversely associated with AD risk, indicating that PTGDR2 upregulation via DNAm reduces AD susceptibility (**Figure 5D**). Lastly, the DNAm probe cg05092371 (OR = 0.97, [95% CI: 0.95–1.00], FDR = 3.87 × 10^−^²) was associated with reduced AD risk through positive regulation of CD28 expression (OR = 0.64, [95% CI: 0.46–0.89], FDR = 2.73 × 10^−^²) (**Figure 5E**).

### Cell type-specific expression in peripheral blood in AD

**Figure 6:**
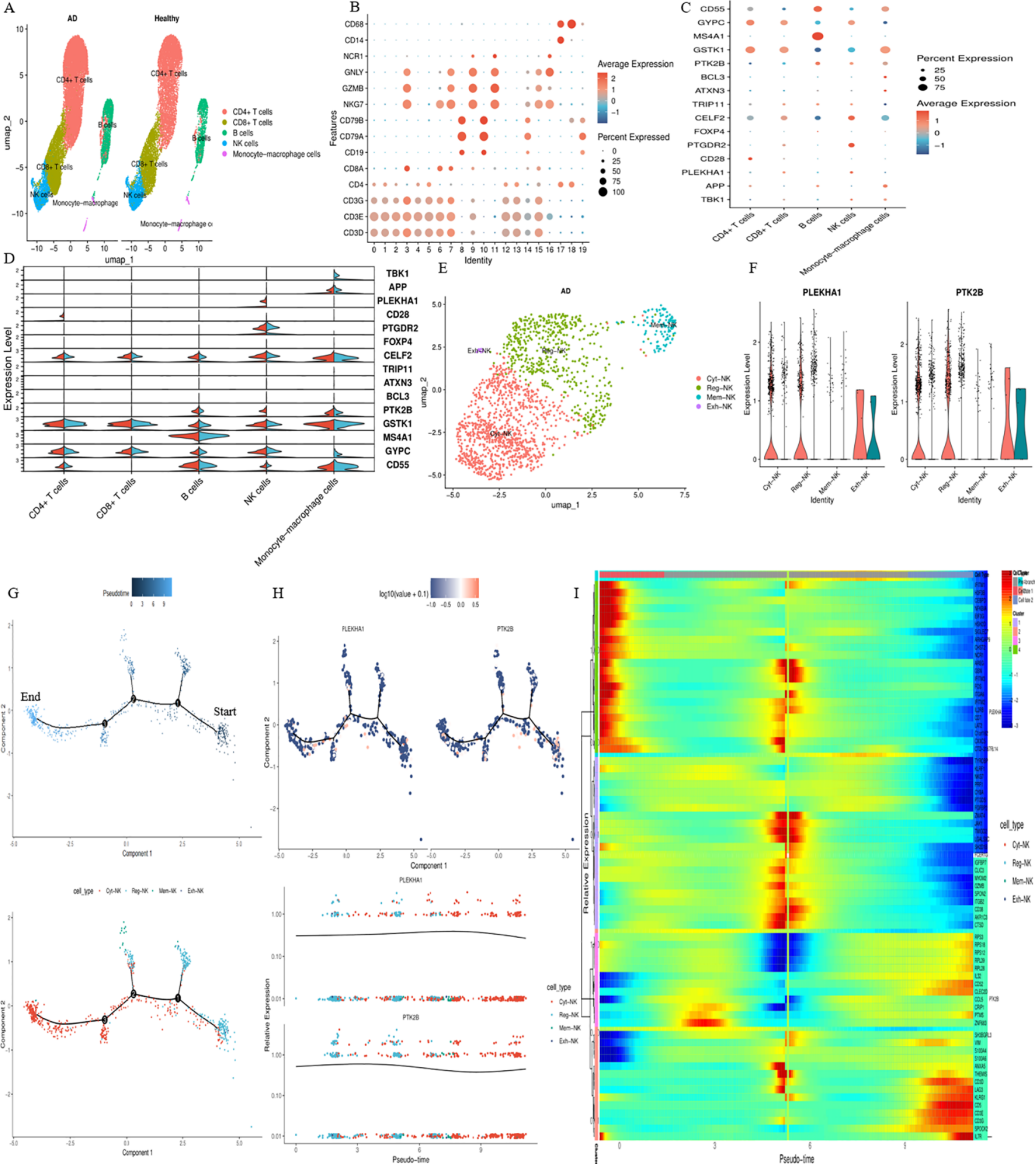
Single-cell type-specific expression profiling results. **(A)** Visualisation of cell annotation results based on UMAP clustering in normal and AD groups. **(B)** Expression patterns of marker genes across distinct clusters. **(C)** Single-cell expression profiles of 15 marker genes within different cellular clusters. **(D)** Differential expression analysis of marker genes between the two groups. **(E)** Clustering outcomes of NK cells. **(F)** Disparate expression patterns of PLEKHA1 and PTK2B genes among NK cell subtypes. **(G)** Pseudotemporal trajectory of single-cell transcriptomic data with cluster-specific colouration. **(H)** Temporal trajectory depicting pseudotemporal expression dynamics of PLEKHA1 and PTK2B genes. **(I)** Expression kinetics and aggregation of representative genes along the pseudotemporal progression of NK cells, with gene nomenclature and corresponding cell types annotated on the right margin of the heatmap.

To determine whether the five protein-coding genes and ten previously identified AD-associated variant genes display cell-type-specific enrichment in AD peripheral blood, scRNA-seq data from the GEO database were analysed. Unsupervised clustering of the discovery dataset partitioned peripheral immune cells into 20 subclusters, subsequently annotated into five major cell types (**Figures 6A, B**), and heatmap visualization confirmed distinct expression patterns of 15 marker genes across clusters (**Figure 6C**). Differential expression analysis identified cell-type-specific enrichment of three protein-coding genes in AD patients: CD28 in CD4+ T cells (log2FC > 1, FDR < 0.05), and PLEKHA1 and PTK2B, which were enriched in NK cells among lymphocyte populations (**Figure 6D**). In healthy controls, TBK1 and GYPC showed preferential expression in monocyte-macrophage cells. NK cells were then subsetted and reclustered into four functionally distinct subpopulations (**Figure 6E**). PLEKHA1 and PTK2B showed predominant coexpression in Cyt-NK and Reg-NK subpopulations (**Figure 6F**). Pseudo-time trajectory analysis revealed a three-branched developmental architecture, with distinct clustering of NK subpopulations along transcriptional-state paths (**Figure 6G**). Projection of PLEKHA1 and PTK2B expression onto this trajectory showed that Reg-NK cells had low baseline PLEKHA1 levels in early pseudotime states with progressive upregulation. In contrast, Cyt-NK cells maintained sustained high expression of both genes, consistent with involvement in cytoskeletal reorganization and cytotoxic signalling (**Figure 6H**). Mem-NK and Exh-NK subsets retained relatively stable, low-level expression. Heatmap analysis across pseudotime (**Figure 6I**) revealed NK subtype-specific clustering patterns and co-regulated gene modules, with PLEKHA1 and PTK2B co-expression in Reg-NK and Cyt-NK at specific pseudotime phases, reinforcing their coordinated roles in NK-mediated immune regulation.

These discovery-phase findings were tested in the independent GSE226602 validation cohort. Unsupervised clustering identified 19 cell types (**Supplementary Figures S12A, B**). Expression profiling of six core candidate genes confirmed that PLEKHA1 was most highly expressed in NK cells among lymphocyte populations, PTK2B showed enrichment in both NK cells and CD14 Monocytes, and CD28 was predominantly expressed in Treg and CD4 TCM cells (**Supplementary Figure S12C**), all consistent with the discovery cohort. Differential expression analysis within each cell type yielded 19 significant associations (adjusted P < 0.05): BCL3 was upregulated in CD14 Monocytes (log2FC = 0.42, P_adj = 2.18 × 10^−28^), CD55 was downregulated in CD4 Naive (log2FC = −0.20, P_adj = 2.41 × 10^−41^) and B naive cells (log2FC = −0.22, P_adj = 8.44 × 10^−^¹²), CD28 was upregulated in CD8 TEM cells (log2FC = 0.088, P_adj = 1.09 × 10^−^³³), and GSTK1 was consistently downregulated across CD14 Monocytes, CD8 TCM, and CD4 Naive populations (**Supplementary Figure S12D**). Sub-clustering of NK cells (n = 33,078; AD = 22,169, HC = 10,909) resolved nine functional subtypes (**Supplementary Figures S13A, B**). Compositional analysis uncovered pronounced differences: Regulatory NK cells were almost exclusively present in AD patients (AD: 985 vs HC: 4; OR = 126.4, P_adj = 3.51 × 10^−165^), Early-activated NK cells were similarly enriched in AD (AD: 371 vs HC: 8; OR = 23.2, P_adj = 9.78 × 10^−5^³), and Activated NK cells were depleted (AD: 14.9% vs HC: 24.3%; OR = 0.55, P_adj = 2.06 × 10^−91^) (**Supplementary Figure S13C**). PLEKHA1 and PTK2B were expressed across all NK subtypes (**Supplementary Figures S13D, E**), though neither gene reached significant differential expression between AD and HC within individual subtypes. The absence of within-subtype transcriptional differences, alongside the dramatic compositional remodelling of the NK compartment, suggests that AD-associated NK cell dysfunction operates primarily through shifts in subtype proportions rather than through altered gene expression within a given subtype. We interrogated the DSigDB for compounds targeting PLEKHA1/PTK2B-associated pathways to explore therapeutic implications. **Supplementary Table S13** summarizes the mechanisms of potential drugs targeted by drug-ready genes and their possible use in AD treatment.

### TWAS analysis

The cross-tissue TWAS analysis identified 86 genes with FDR< 0.05 (**Supplementary Table 14**). COJO analysis was conducted on candidate genes in whole-blood samples to eliminate false-positive results arising from LD. The results indicated that three genes still exhibited significant, independent genetic signals in whole-blood tissue (**Supplementary Table S15**). For the genes PLEKHA1 (FDR = 2.10×10^−4^), PTK2B (FDR = 2.50×10^−4^), and TBK1 (FDR = 2.30×10^−4^), their association signals with AD remained highly significant after the COJO analysis (**Supplementary Figures S14**–**S16**).

### Cohort validation results

**Figure 7:**
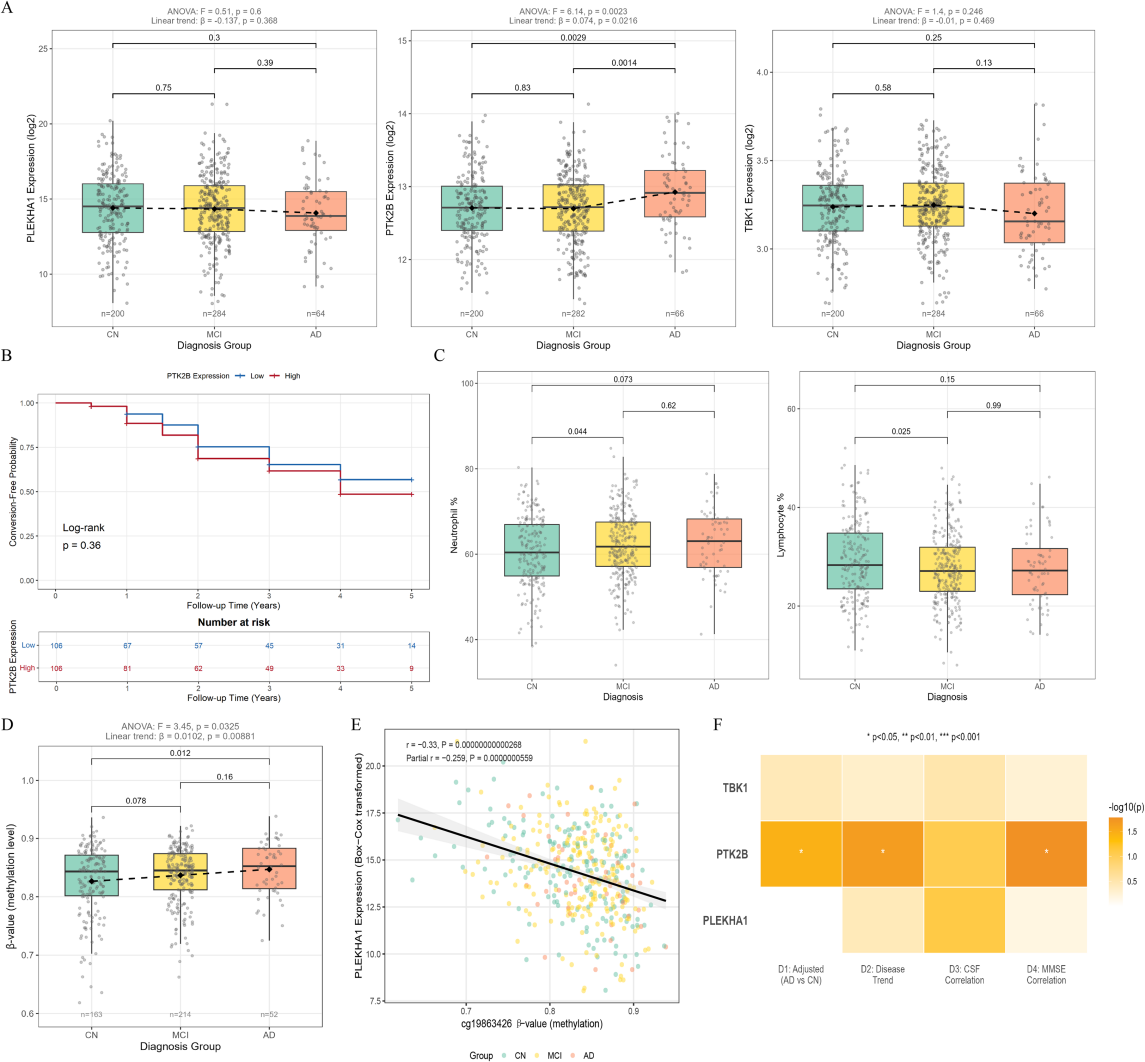
Independent clinical cohort validation of candidate genes in the ADNI cohort (N = 550). **(A)** Box plots of peripheral blood gene expression levels for PLEKHA1, PTK2B, and TBK1 across CN (n = 200), MCI (n = 284), and AD (n = 66) groups. Dashed lines connect group means. ANOVA F-statistics and linear trend coefficients are shown above each panel. Pairwise P-values are from two-sample t-tests. **(B)** Kaplan-Meier curves for MCI-to-AD conversion stratified by median PTK2B expression (N = 214 baseline MCI, 64 converters within 5 years). **(C)** Box plots of peripheral blood neutrophil percentage (left) and lymphocyte percentage (right) across diagnostic groups. Pairwise P-values are from two-sample t-tests. **(D)** Box plot of cg19863426 (PLEKHA1 promoter) DNA methylation beta-values across diagnostic groups (n = 163 CN, 214 MCI, 52 AD with available methylation data). **(E)** Scatter plot of cg19863426 methylation versus PLEKHA1 mRNA expression (Box-Cox transformed). **(F)** Heatmap summarizing the four-dimensional validation results for PLEKHA1, PTK2B, and TBK1. Colour intensity represents −log10(P-value). Asterisks denote significance levels: *P < 0.05, **P < 0.01, ***P < 0.001.

After applying longitudinal diagnostic stability criteria, 550 ADNI participants were retained (200 CN, 284 MCI, 66 AD) (**Supplementary Table S16**). The groups differed in education (P = 0.024), MMSE (CN: 29.0 ± 1.1; MCI: 27.1 ± 1.8; AD: 23.3 ± 2.0; P < 0.001), and APOE ϵ4 carrier frequency (CN: 27.0%; MCI: 46.5%; AD: 57.6%; P < 0.001). CSF biomarkers followed the expected pattern, with lower Aβ42 and higher tau and p-tau in AD (all P < 0.001). Full demographics are presented in **Table 2**, and *post-hoc* comparisons are presented in **Supplementary Table S17**. In covariate-adjusted regression controlling for age, sex, and APOE ϵ4, PTK2B expression was elevated in AD relative to CN (β = 0.149, 95% CI 0.011 to 0.287, P = 0.034), while PLEKHA1 (β = 0.044, P = 0.530) and TBK1 (β = 0.024, P = 0.710) showed no significant group differences (**Supplementary Figure S17**). One-way ANOVA confirmed a significant group effect for PTK2B (F = 6.14, P = 0.0023) with a monotonic increase across the CN-MCI-AD continuum (linear trend β = 0.089, P = 0.0006), whereas PLEKHA1 (P = 0.31) and TBK1 (P = 0.42) did not reach significance (**Figure 7A**). Partial correlation adjusting for age, sex, and APOE ϵ4 revealed a positive association between PLEKHA1 expression and CSF total tau (partial r = 0.102, P = 0.036) (**Supplementary Figure S18**), while PTK2B showed a trend-level inverse correlation with CSF Aβ42 (partial r = −0.078, P = 0.11) (**Supplementary Figure S19**). TBK1 was not significantly associated with any CSF marker. PTK2B expression was inversely correlated with MMSE (Pearson r = −0.164, P = 0.017), and this association persisted after covariate adjustment. PLEKHA1 and TBK1 showed no significant cognitive correlations (**Supplementary Figure S20**).

**Table 2:** Baseline demographics and clinical characteristics of the ADNI validation cohort (N = 550).

Characteristic	Total (N = 550)	CN (n = 200)	MCI (n = 284)	AD (n = 66)	P value	Test
Age, years (mean ± SD)	75.4 ± 6.8	75.8 ± 5.4	74.7 ± 7.5	77.7 ± 7.5	0.003	ANOVA
Female, n (%)	242 (44.0%)	106 (53.0%)	116 (40.8%)	20 (30.3%)	0.002	Chi-square
Education, years (mean ± SD)	16.0 ± 2.8	16.4 ± 2.7	15.9 ± 2.9	15.3 ± 3.0	0.024	ANOVA
APOE ϵ4 carrier, n (%)	216 (39.3%)	54 (27.0%)	132 (46.5%)	38 (57.6%)	<0.001	Chi-square
MMSE score (mean ± SD)	27.4 ± 2.7	29.0 ± 1.1	27.1 ± 1.8	23.3 ± 2.0	<0.001	ANOVA
ADAS-Cog score (mean ± SD)	8.7 ± 5.5	5.5 ± 2.8	9.1 ± 4.3	19.3 ± 5.8	<0.001	ANOVA
CDR-SB (median, IQR)	1.0 (0.0-4.0)	0.0 (0.0-0.0)	1.5 (0.5-2.0)	5.5 (4.5-8.0)	<0.001	Kruskal-Wallis
RAVLT Immediate (mean ± SD)	38.1 ± 12.9	46.1 ± 10.5	36.5 ± 10.4	19.8 ± 7.5	<0.001	ANOVA
ADNI-MEM composite (mean ± SD)	0.51 ± 1.01	1.22 ± 0.67	0.38 ± 0.72	-1.22 ± 0.68	<0.001	ANOVA
ADNI-EF composite (mean ± SD)	0.39 ± 0.98	0.87 ± 0.79	0.32 ± 0.84	-0.89 ± 0.93	<0.001	ANOVA
CSF Aβ42, pg/mL (mean ± SD)	1144.6 ± 597.3	1364.4 ± 636.6	1073.2 ± 547.7	714.9 ± 354.7	<0.001	ANOVA
CSF t-Tau, pg/mL (mean ± SD)	268.6 ± 105.2	245.5 ± 83.8	275.0 ± 111.6	322.0 ± 118.8	<0.001	ANOVA
CSF p-Tau, pg/mL (mean ± SD)	24.9 ± 10.9	22.6 ± 9.1	25.7 ± 11.6	29.7 ± 11.2	<0.001	ANOVA
t-Tau/Aβ42 ratio (mean ± SD)	0.305 ± 0.212	0.228 ± 0.157	0.319 ± 0.210	0.533 ± 0.243	<0.001	ANOVA
p-Tau/Aβ42 ratio (mean ± SD)	0.029 ± 0.023	0.021 ± 0.017	0.031 ± 0.023	0.053 ± 0.026	<0.001	ANOVA
WBC, 10³/μL (mean ± SD)	6.46 ± 1.59	6.41 ± 1.57	6.50 ± 1.62	6.45 ± 1.55	0.817	ANOVA
Neutrophil % (mean ± SD)	61.7 ± 8.0	60.7 ± 8.3	62.2 ± 7.7	62.7 ± 7.7	0.068	ANOVA
Lymphocyte % (mean ± SD)	28.2 ± 7.5	29.3 ± 8.0	27.7 ± 7.1	27.6 ± 7.5	0.058	ANOVA
Monocyte % (mean ± SD)	6.4 ± 1.8	6.4 ± 1.7	6.5 ± 1.8	6.1 ± 1.7	0.193	ANOVA
PLEKHA1 expression (mean ± SD)	5.43 ± 0.43	5.44 ± 0.43	5.43 ± 0.43	5.38 ± 0.42	0.616	ANOVA
PTK2B expression (mean ± SD)	8.14 ± 0.21	8.13 ± 0.21	8.12 ± 0.21	8.22 ± 0.22	0.002	ANOVA
TBK1 expression (mean ± SD)	6.05 ± 0.43	6.05 ± 0.43	6.07 ± 0.42	5.97 ± 0.48	0.259	ANOVA

Among 214 baseline MCI participants (64 converters within 5 years), Cox regression adjusted for age and sex identified PTK2B as a predictor of MCI-to-AD conversion (HR = 1.741, 95% CI 1.000 to 3.030, P = 0.050), an effect that was attenuated but directionally consistent after further APOE ϵ4 adjustment. Kaplan-Meier curves stratified by median PTK2B expression showed earlier conversion in the high-expression group (**Figure 7B**). PLEKHA1 and TBK1 did not predict conversion risk. Standardized regression revealed strong associations between candidate gene expression and peripheral immune cell proportions: PLEKHA1 was positively associated with lymphocyte percentage (standardized β = 0.350, P < 10^−15^) and inversely with neutrophil percentage (standardized β = −0.308, P < 10^−^¹²), while PTK2B showed the opposite pattern (neutrophil β = 0.236, P < 10^−7^, lymphocyte β = −0.241, P < 10^−7^). These immune proportions were themselves linked to CSF amyloid pathology: neutrophil percentage was inversely associated with Aβ42 (standardized β = −0.102, P = 0.035), and lymphocyte percentage was positively associated (standardized β = 0.105, P = 0.031), linking peripheral immune shifts to central amyloid burden (**Figure 7C**; **Supplementary Table S18**). In the 144 participants with plasma proteomic data, no gene-protein associations survived FDR correction, consistent with the limited sample size. At the nominal level, PLEKHA1 was associated with HGF (P = 0.002), A1Micro (P = 0.002), TNF-α (P = 0.004), and B2M (P = 0.006), proteins involved in inflammatory and immune signalling. ANCOVA identified group differences for A1Micro (P = 0.0003) and PYY (P = 0.003). These proteomic results are reported as hypothesis-generating (**Supplementary Tables S19**, **S20**).

Of the 550 participants, 429 (78.0%) had whole-genome DNA methylation data (Illumina HumanMethylation450 BeadChip, Noob normalization). For the SMR-prioritized probe cg19863426 at the PLEKHA1 promoter (~4.6 kb upstream of the TSS), beta-values increased across the CN-MCI-AD spectrum (CN 0.826 ± 0.061, MCI 0.837 ± 0.048, AD 0.847 ± 0.047, ANOVA F = 3.45, P = 0.032, linear trend β = 0.010, P = 0.009), with Tukey *post-hoc* testing confirming higher methylation in AD than CN (P = 0.042) (**Figure 7D**; **Supplementary Table S21**). This hypermethylation correlated inversely with PLEKHA1 mRNA (Pearson r = −0.33, P = 2.68 × 10^−^¹², partial r = −0.33 adjusted for age, sex, and APOE ϵ4) (**Figure 7E**; **Supplementary Table S22**), directly supporting the SMR-predicted mechanism in which promoter methylation suppresses PLEKHA1 transcription. Methylation at cg19863426 was also associated with peripheral immune composition (neutrophil percentage β = 0.311, P = 4.3 × 10^−10^, lymphocyte percentage β = −0.343, P = 3.9 × 10^−^¹²) (**Supplementary Table S23**), a pattern opposite to that of PLEKHA1 mRNA and consistent with methylation-mediated transcriptional suppression shifting the neutrophil-to-lymphocyte balance. Covariate-adjusted regression linking cg19863426 to CSF biomarkers yielded no significant associations (**Supplementary Table S24**). Unadjusted correlations with RAVLT immediate recall (r = −0.190, P = 8.3 × 10^−5^) and ADNI-MEM (r = −0.165, P = 6.4 × 10^−4^) were attenuated after adjustment (P = 0.075 and P = 0.21), suggesting partial confounding by age and APOE ϵ4 (**Supplementary Table S25**). Sex and age subgroup analyses for cg19863426 are presented in **Supplementary Figure S21**. The intragenic TBK1 probe cg16604658 showed no group differences (P = 0.65) and was uncorrelated with TBK1 expression (P = 0.84) (**Supplementary Figure S22**). Among five extended SMR probes, cg14130459 (PTGDR2) showed a significant linear decline across disease stages (trend β = −0.014, P = 0.026), with the AD group lower than the CN group (t-test, P = 0.031). However, the omnibus ANOVA did not reach significance (F = 2.84, P = 0.060) (**Supplementary Figure S23**), Tukey AD vs CN P = 0.047) and cg19788250 (APP) a MCI-AD difference (Tukey P = 0.013) (**Supplementary Figure S24**). In comparison, the remaining three probes did not reach significance (**Supplementary Figures S25**–**S27**). Immune associations paralleling the cg19863426 pattern were observed for cg14130459 and two APP probes.

Sex-stratified analysis showed that the PTK2B AD versus CN effect was significant in males (β = 0.246, P = 0.006) but not in females (β = 0.069, P = 0.476), though the Group × Sex interaction did not reach significance. In participants aged ≥75 years, PTK2B showed a trend-level group difference (P = 0.074). PLEKHA1 and TBK1 had no significant subgroup effects (**Supplementary Figure S28**). The PTK2B finding was robust to IQR-based outlier exclusion (ANOVA F = 8.03, P = 0.005) and Bonferroni correction across all six primary tests (corrected P = 0.014). APOE ϵ4-stratified analyses confirmed group differences in both carrier and non-carrier subgroups (**Supplementary Tables S26**, **S27**). In the AddNeuroMed cohort (N = 716, 240 CN, 192 MCI, 284 AD), ANCOVA adjusted for age and sex replicated the PTK2B elevation in AD (T = 3.87, FDR P = 3.56 × 10^−4^). PLEKHA1 showed a nominally significant MCI versus CN difference (T = 2.11, P = 0.036, FDR P = 0.225), and TBK1 did not reach significance (**Supplementary Table S28**). Across the six validation dimensions, PTK2B was the most consistently supported candidate, with significant disease-stage differences in both cohorts (ADNI P = 0.0023, AddNeuroMed FDR P = 3.56 × 10^−4^), cognitive correlation (MMSE r = −0.164, P = 0.017), conversion prediction (HR = 1.741, P = 0.050), and immune cell associations (P < 10^−7^). PLEKHA1 showed a distinct profile of CSF tau correlation (partial r = 0.102, P = 0.036), immune cell associations (P < 10^−^¹²), and nominal proteomic links to HGF, TNF-α, and B2M, without significant group-level expression differences. TBK1 did not show consistent associations in peripheral blood. The four-dimensional validation heatmap is shown in **Figure 7F**.

## Discussion

This study assembled a multi-omics causal inference pipeline spanning GWAS meta-analysis of 894,710 individuals, blood-based cis-eQTL/cis-mQTL integration via three-step SMR, scRNA-seq profiling, TWAS, and independent cohort validation in the ADNI cohorts, alongside real Illumina HumanMethylation450 BeadChip data from 429 participants. The convergence of genetic, epigenetic, transcriptomic, and clinical evidence on PTK2B and PLEKHA1 supports the view that peripheral immune gene dysregulation is mechanistically coupled to AD neuropathology, rather than representing a passive correlate of disease status. PTK2B showed the most complete validation chain. Mendelian randomization identified a positive causal effect on AD risk, TWAS confirmed an independent signal after conditional joint analysis, and the ADNI cohort demonstrated monotonically increasing expression across the CN-MCI-AD spectrum. This trajectory was further supported by an inverse correlation with MMSE scores and prediction of MCI-to-AD conversion in Cox regression, with cross-cohort replication in AddNeuroMed. These results are consistent with functional studies showing that PTK2B/Pyk2 overexpression exacerbates amyloid pathology in transgenic models and that PTK2B maps to a replicated AD risk locus at 8p21.1 (32). The sex-stratified pattern, with a stronger effect in males, aligns with emerging evidence of sex-differential genetic architecture in AD and merits investigation in larger sex-balanced samples (33).

PLEKHA1 presented a different but equally informative validation pattern. Bulk mRNA levels did not differ across diagnostic groups. Yet, the SMR-prioritized probe cg19863426 at the PLEKHA1 promoter showed progressive hypermethylation from CN to AD and was strongly inversely correlated with PLEKHA1 mRNA, directly validating the SMR-predicted regulatory mechanism. The apparent discrepancy between non-significant mRNA group differences and significant epigenetic changes likely reflects a confound due to cell-type composition. scRNA-seq localized PLEKHA1 to NK cells, which constitute only 5-15% of peripheral blood mononuclear cells, suggesting that disease-associated transcriptional changes in this minority population are diluted in bulk assays. This interpretation is reinforced by the opposing immune cell associations of PLEKHA1 mRNA and cg19863426 methylation, consistent with a model in which promoter hypermethylation suppresses PLEKHA1 transcription and shifts the neutrophil-to-lymphocyte balance. The scRNA-seq validation cohort revealed profound compositional remodelling, with Regulatory NK cells almost exclusively present in AD and Activated NK cells depleted. The correlation between PLEKHA1 expression and CSF total tau positions this gene as a molecular node linking peripheral epigenetic regulation to central tau pathology (34). Among extended SMR targets, PTGDR2 showed partial epigenetic validation, while drug signature analysis identified compounds targeting PLEKHA1/PTK2B pathways, including citric acid, offering hypothesis-generating pharmacological leads.

The TBK1 results require particular scrutiny, as they appear to contradict the SMR prediction and the cohort validation data. Both SMR and TWAS supported a causal role for TBK1 in AD. Yet, neither TBK1 mRNA nor cg16604658 methylation showed group differences in the ADNI cohort, and scRNA-seq revealed lower TBK1 expression specifically in AD monocyte-macrophages. Two complementary explanations account for this discrepancy. The first is a whole-blood dilution effect. TBK1 expression was concentrated in monocyte-macrophages, which represent 5-10% of circulating leukocytes, rendering transcriptional changes below the detection threshold of bulk assays. The second explanation addresses a directional paradox that warrants explicit discussion. The SMR result indicates that genetically elevated TBK1 expression increases AD risk (OR = 1.25), yet scRNA-seq showed reduced TBK1 expression in AD monocyte-macrophages. These two observations are not contradictory when the temporal framework of each method is considered. MR captures the cumulative, lifelong effect of constitutively higher TBK1 expression driven by germline cis-regulatory variants. TBK1 is a central kinase in the STING-TBK1-IRF3 innate immune axis (35, 36), and chronically elevated TBK1 activity would be expected to sustain type I interferon signalling in monocyte-macrophages over decades, promoting a pro-inflammatory milieu that accelerates neuroinflammatory damage. Sub-clustering of the monocyte compartment in the validation cohort (11,365 cells) confirmed that TBK1 showed a consistent trend toward higher expression in AD across Classical and Intermediate subpopulations (avg log2FC = 0.084 and 0.091, respectively), directionally concordant with the MR prediction. The more informative finding was that STAT1, a downstream effector of TBK1-IRF3 signalling, was significantly suppressed in AD Classical (FDR = 0.004) and Intermediate monocytes (FDR = 3.14 × 10^−15^), accompanied by a compositional shift toward Classical and away from Intermediate subsets, indicating impaired monocyte maturation and downstream interferon signalling exhaustion despite preserved TBK1 transcript levels. This two-phase model, in which genetically driven overexpression initiates chronic inflammation that eventually leads to downstream signalling exhaustion, reconciles the MR and scRNA-seq findings and is consistent with evidence that TBK1 loss-of-function mutations independently cause neurodegeneration through defective autophagy and that TBK1-dependent mitophagy is compromised in AD-derived macrophages (37, 38). Distinguishing between the causal (lifelong overexpression) and consequential (disease-stage signalling collapse) phases of TBK1 dysregulation will require longitudinal monocyte-specific transcriptomic profiling across the preclinical-to-clinical AD continuum. The negative bulk ADNI results should therefore not discount TBK1 involvement, but rather underscore the necessity for fluorescence-activated cell sorting of monocyte subsets in future profiling.

A pervasive constraint of this study is the restriction to European-ancestry populations. All primary GWAS sources, the GTEx V8 eQTL panel, and the blood mQTL reference were derived from European individuals. LD architecture differs substantially across ancestries, and European-derived tag SNPs may not efficiently capture the same causal signals in East Asian or African populations. The APOE locus illustrates this challenge, as ϵ4 allele frequency and effect size vary considerably across populations (39). The independent FinnGen R11 sensitivity analysis confirmed concordant associations for PTK2B, CD55, CELF2, and MS4A1, indicating that sample overlap between primary GWAS sources did not materially inflate the core findings. The directional discordance observed for BCL3 and BIN1, however, likely reflects population-specific haplotype effects at regions with extensive LD. Trans-ethnic GWAS meta-analyses incorporating ancestry-specific fine-mapping are needed to evaluate the global applicability of these peripheral immune targets. Another methodological limitation concerns single-cell transcriptomic profiling. Both our discovery and validation scRNA-seq datasets utilized PBMC preparations isolated via Ficoll density gradient centrifugation. This standard protocol physically excludes granulocytes due to their higher buoyant density, thereby preventing our single-cell analysis from capturing neutrophils. Given the strong associations we observed between candidate gene expression and peripheral neutrophil proportions in the ADNI cohort, future studies using whole-blood single-cell or single-nucleus RNA sequencing are imperative to map innate immune effectors in AD comprehensively.

In conclusion, by integrating GWAS meta-analyses, multi-omics Mendelian randomization, single-cell transcriptomics, and multi-dimensional clinical validation, this study identified the PLEKHA1 epigenetic-silencing axis and the PTK2B transcriptomic trajectory as peripheral immune signatures that correlate with CSF pathology, cognitive decline, and immune cell remodelling in AD. These findings clarify the relationship between peripheral immune gene dysregulation and central neuropathological processes, and provide candidate molecular targets for early diagnosis and therapeutic development.

## Data Availability

The original contributions presented in the study are included in the article/**Supplementary Material**. Further inquiries can be directed to the corresponding authors.
