# Supplementary material for: Deciphering the peripheral immune landscape of Alzheimer’s disease through integrated multi-omics research and cohort validation
Source: Front Immunol. 2026 Apr 13;17:1648591. doi: 10.3389/fimmu.2026.1648591 (PMC13111265; doi:10.3389/fimmu.2026.1648591)
Supplement: Supplementary file 1 [file DataSheet1.docx]

Figure S1-S28

Contents

Figure S1. Quantile-quantile (Q-Q) plot of AD GWAS.

Figure S2. The eQTL data of PTK2B gene expression and the result of MR analysis of AD.

Figure S3. The eQTL data of BCL3 gene expression and the result of MR analysis of AD.

Figure S4. The eQTL data of CD55 gene expression and the result of MR analysis of AD.

Figure S5. The eQTL data of CELF2 gene expression and the result of MR analysis of AD.

Figure S6. The eQTL data of FOXP4 gene expression and the result of MR analysis of AD.

Figure S7. The eQTL data of GSTK1 gene expression and the result of MR analysis of AD.

Figure S8. The eQTL data of GYPC gene expression and the result of MR analysis of AD.

Figure S9. The eQTL data of TRIP11 gene expression and the result of MR analysis of AD.

Figure S10. KEGG and GO analysis of AD GWAS variant sites in GTEx V8 blood tissues.

Figure S11. Colocalization analysis of cis-eQTLs and AD GWAS loci.

Figure S12. Independent validation of PBMC single-cell transcriptomics and candidate gene enrichment in the GSE226602 cohort.

Figure S13. High-resolution sub-clustering reveals dramatic compositional remodeling of NK cells in Alzheimer's disease.

Figure S14. Regional association of TWAS hits for PLEKHA1.

Figure S15. Regional association of TWAS hits for PTK2B.

Figure S16. Regional association of TWAS hits for TBK1.

Figure S17. Covariate-adjusted regression of gene expression across diagnostic groups in the ADNI cohort.

Figure S18. Associations between PLEKHA1 expression and CSF biomarkers in the ADNI cohort (N = 425).

Figure S19. Associations between PTK2B expression and CSF biomarkers in the ADNI cohort (N = 425).

Figure S20. Associations between PLEKHA1, PTK2B, and TBK1 expression and cognitive function (MMSE) in the ADNI cohort (N = 425).

Figure S21. Subgroup analyses of cg19863426 methylation β-values across the cognitively normal (CN), mild cognitive impairment (MCI), and Alzheimer’s disease (AD) diagnostic spectrum.

Figure S22. Subgroup analyses of cg16604658 methylation β-values across the cognitively normal (CN), mild cognitive impairment (MCI), and Alzheimer’s disease (AD) diagnostic spectrum.

Figure S23. Subgroup analyses of cg14130459 (PTGDR2) methylation β-values across the cognitively normal (CN), mild cognitive impairment (MCI), and Alzheimer’s disease (AD) diagnostic spectrum.

Figure S24. Subgroup analyses of cg19788250 (APP) methylation β-values across the cognitively normal (CN), mild cognitive impairment (MCI), and Alzheimer’s disease (AD) diagnostic spectrum.

Figure S25. Subgroup analyses of cg01286133 (APP) methylation β-values across the cognitively normal (CN), mild cognitive impairment (MCI), and Alzheimer’s disease (AD) diagnostic spectrum.

Figure S26. Subgroup analyses of cg18597421 (APP) methylation β-values across the cognitively normal (CN), mild cognitive impairment (MCI), and Alzheimer’s disease (AD) diagnostic spectrum.

Figure S27. Subgroup analyses of cg05092371 (CD28) methylation β-values across the cognitively normal (CN), mild cognitive impairment (MCI), and Alzheimer’s disease (AD) diagnostic spectrum.

Figure S28. Subgroup analysis of AD-associated gene methylation effects on AD risk (AD vs CN, adjusted).


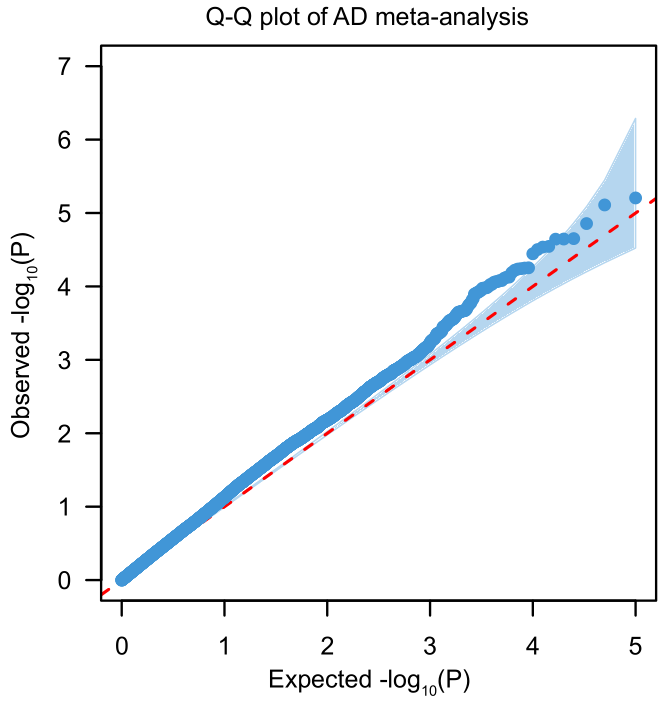


Figure S1. Quantile-quantile (Q-Q) plot of AD GWAS. The Q-Q plot demonstrates the number and magnitude of observed associations between genotyped SNPs and AD compared to the expected association statistics under the null hypothesis that there is no association. The identity line is shown in red. Observed association statistics (y-axis) and expected association statistics (x-axis) are on a 𝑙𝑜𝑔 𝑃 scale.


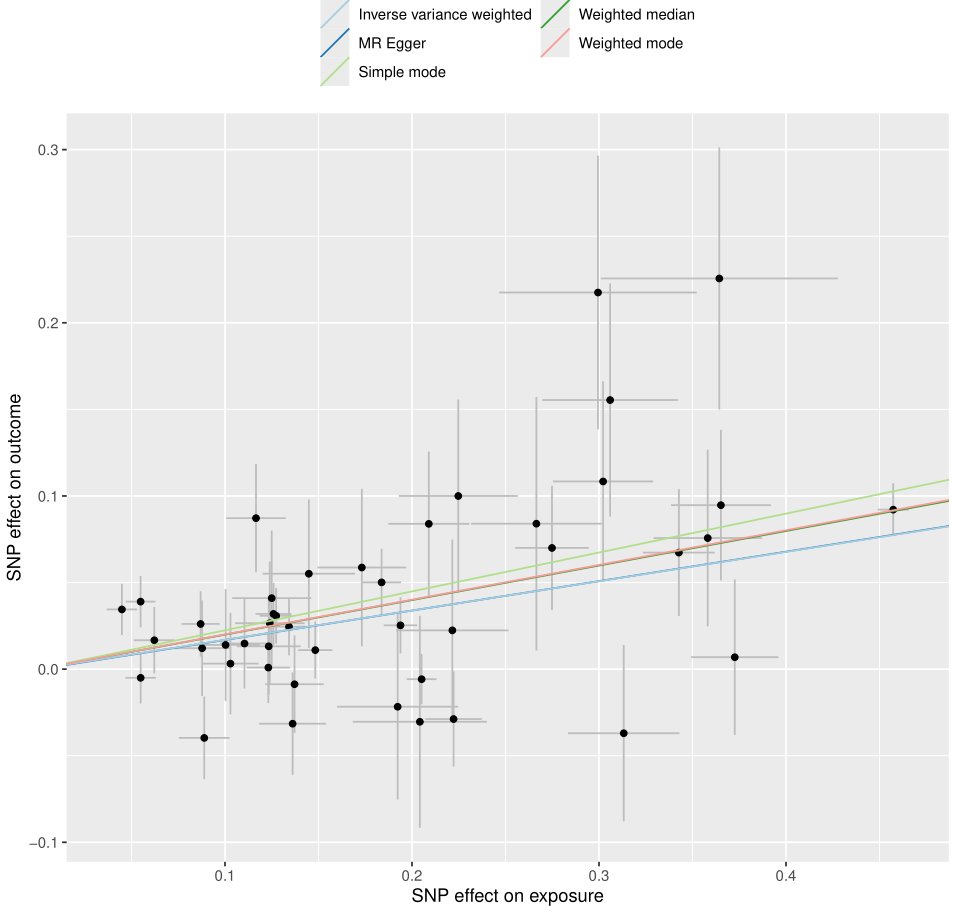

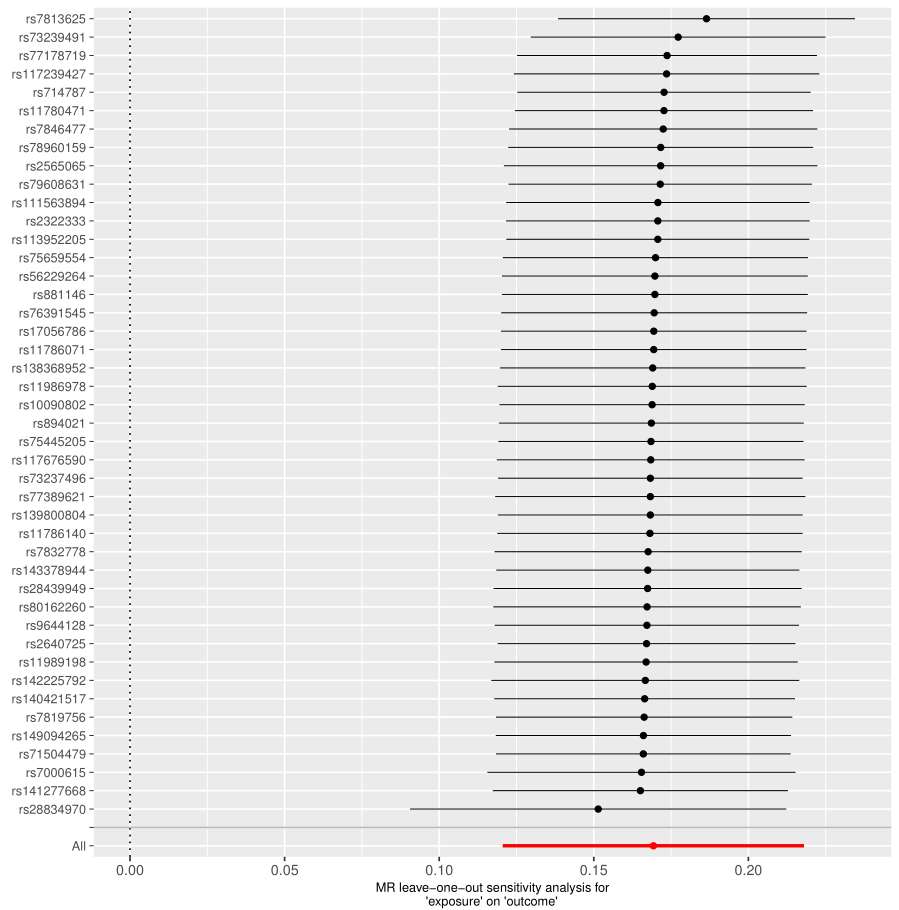


Figure S2. The eQTL data of PTK2B gene expression and the result of MR analysis of AD.


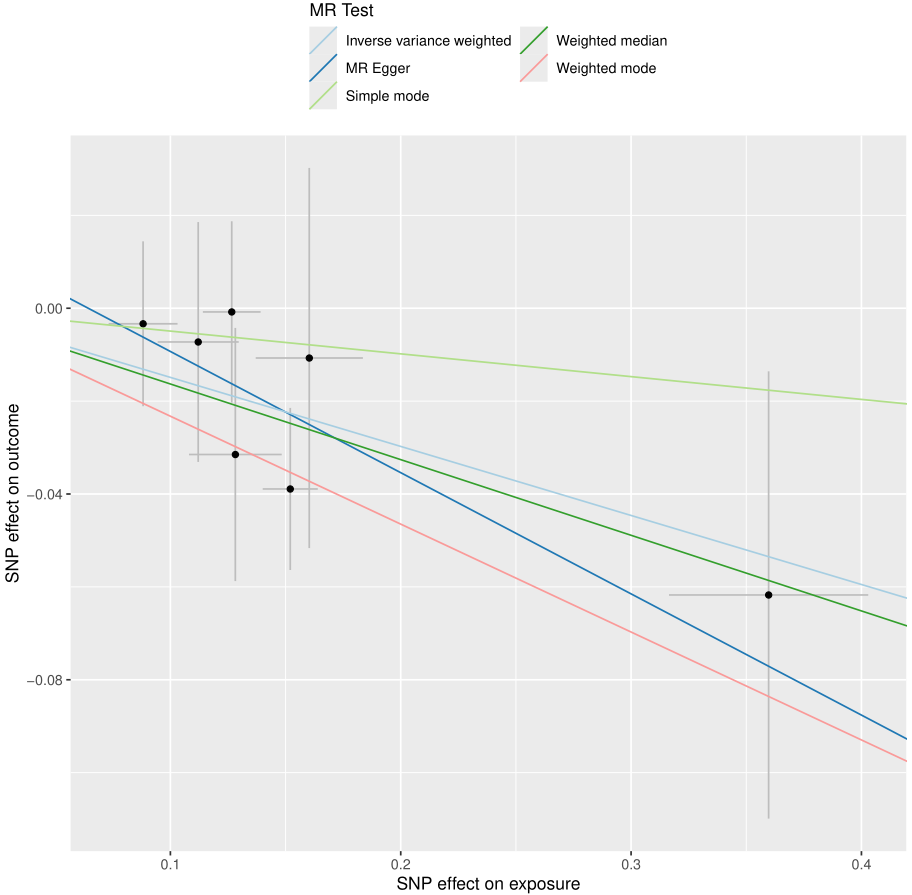


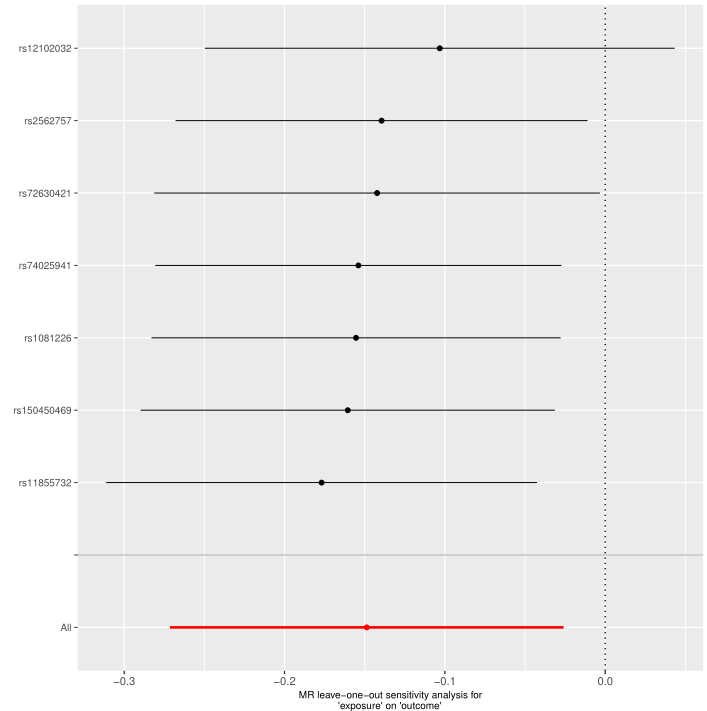


Figure S3. The eQTL data of BCL3 gene expression and the result of MR analysis of AD.


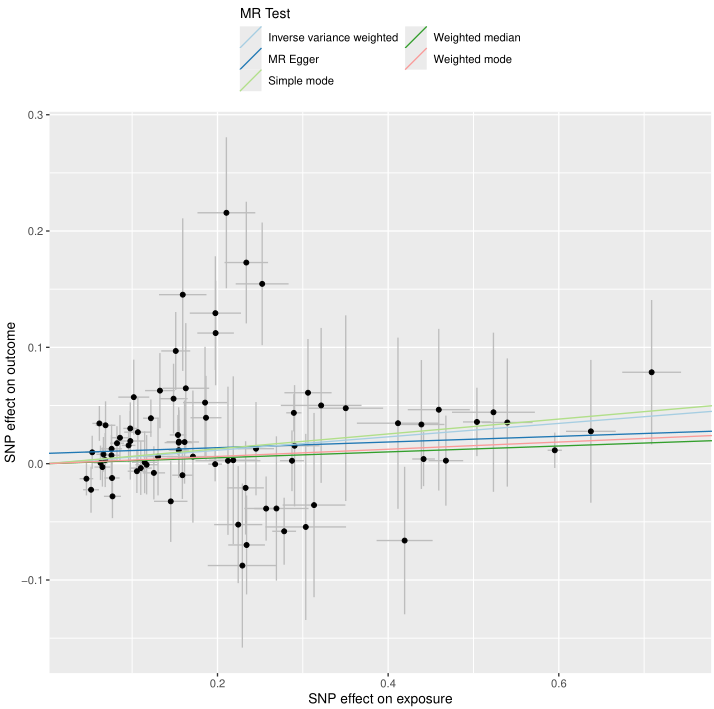


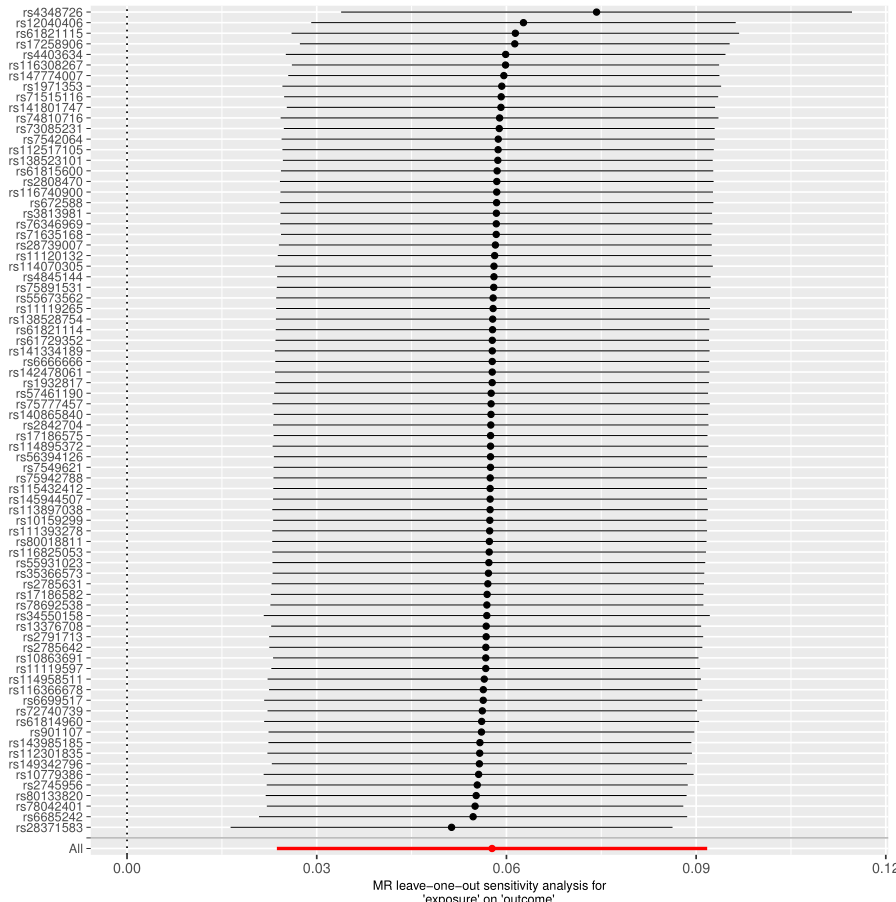


Figure S4. The eQTL data of CD55 gene expression and the result of MR analysis of AD.


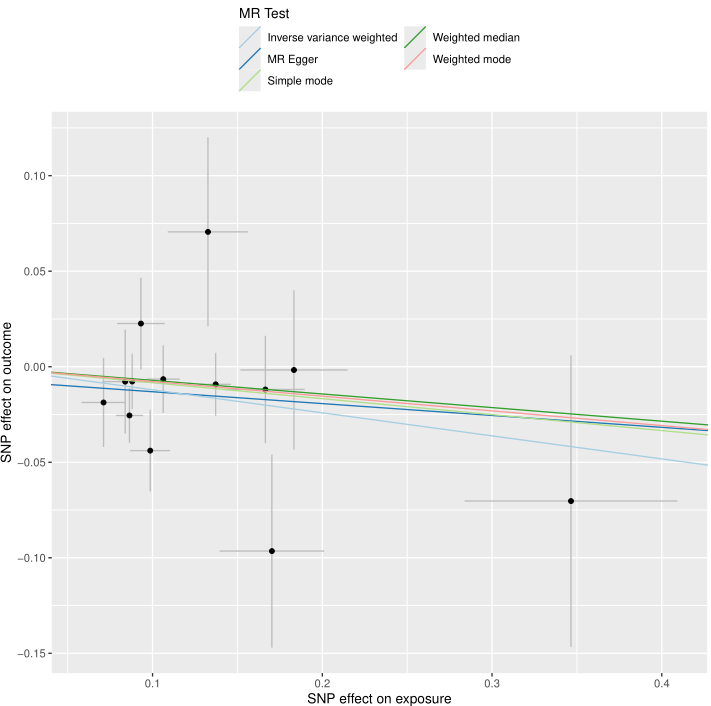


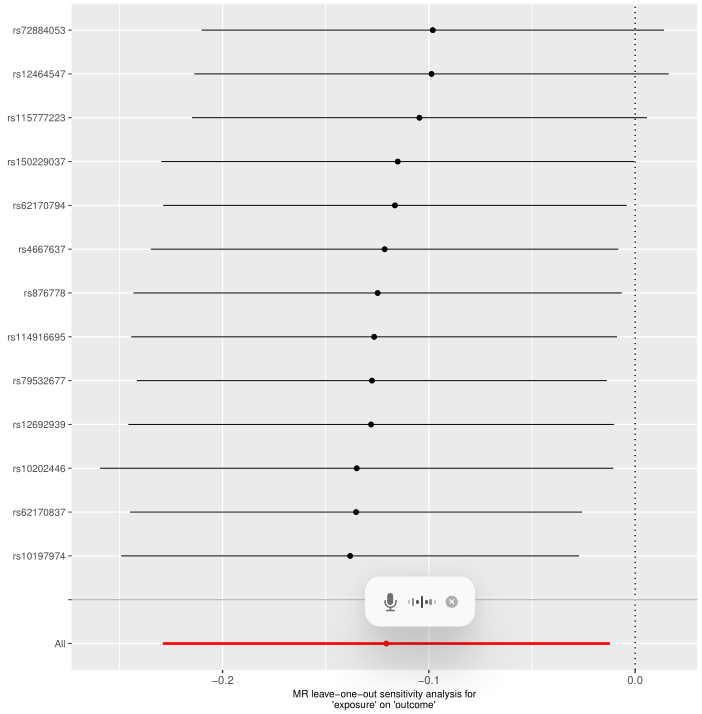


Figure S5. The eQTL data of CELF2 gene expression and the result of MR analysis of AD.


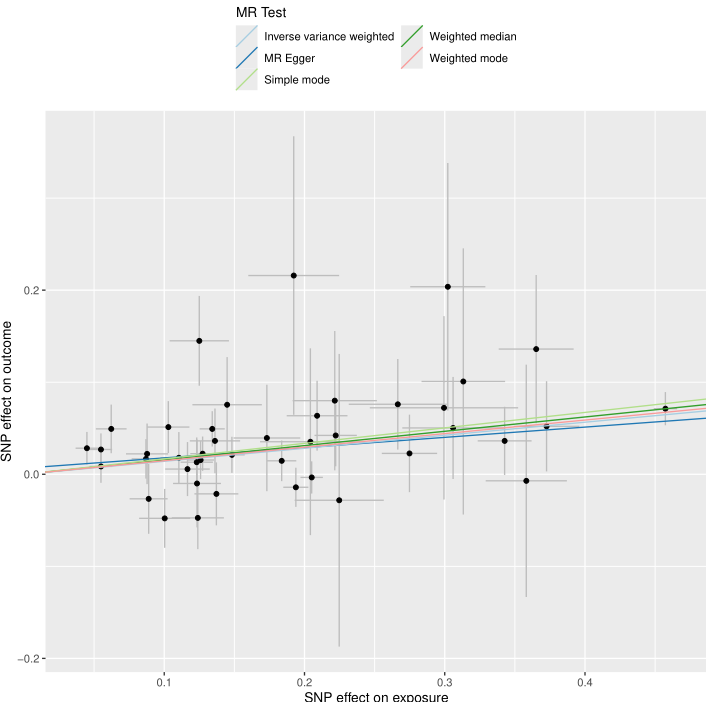


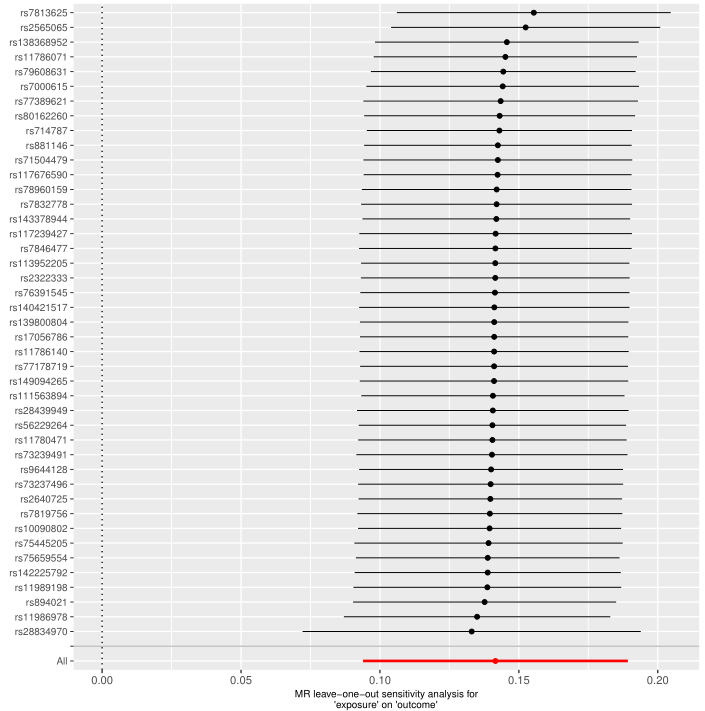


Figure S6. The eQTL data of FOXP4 gene expression and the result of MR analysis of AD.


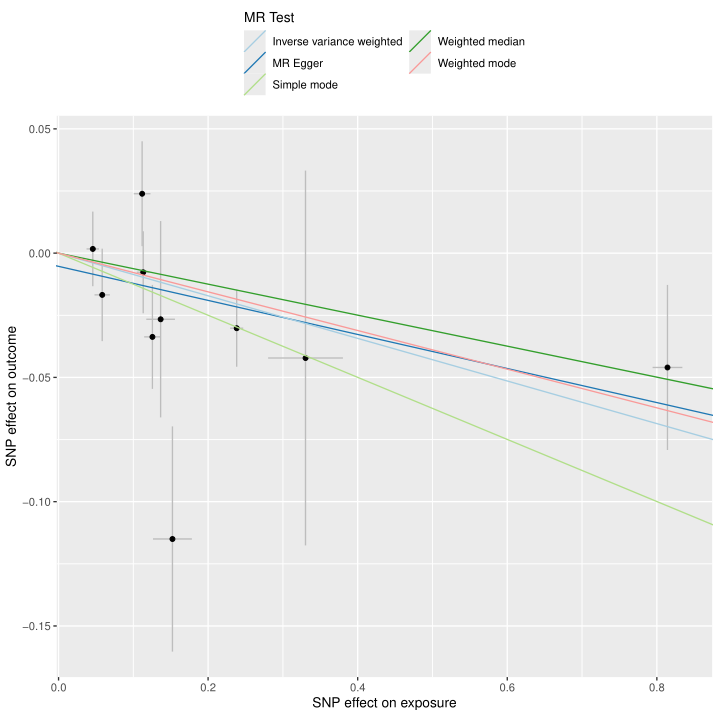


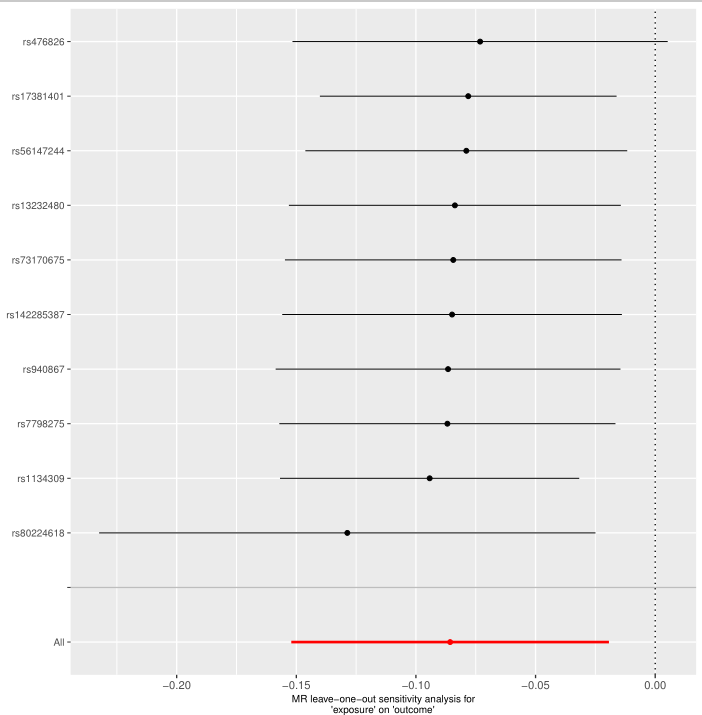


Figure S7. The eQTL data of GSTK1 gene expression and the result of MR analysis of AD.


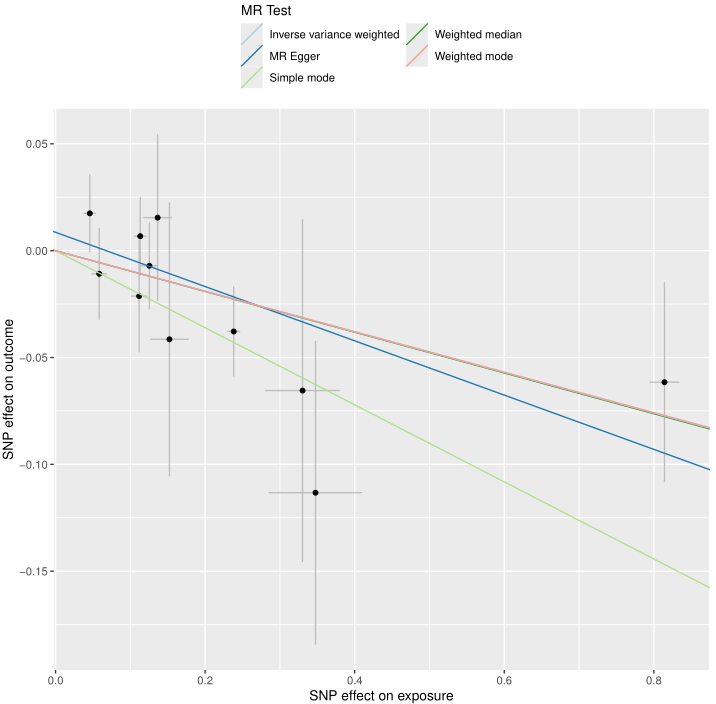


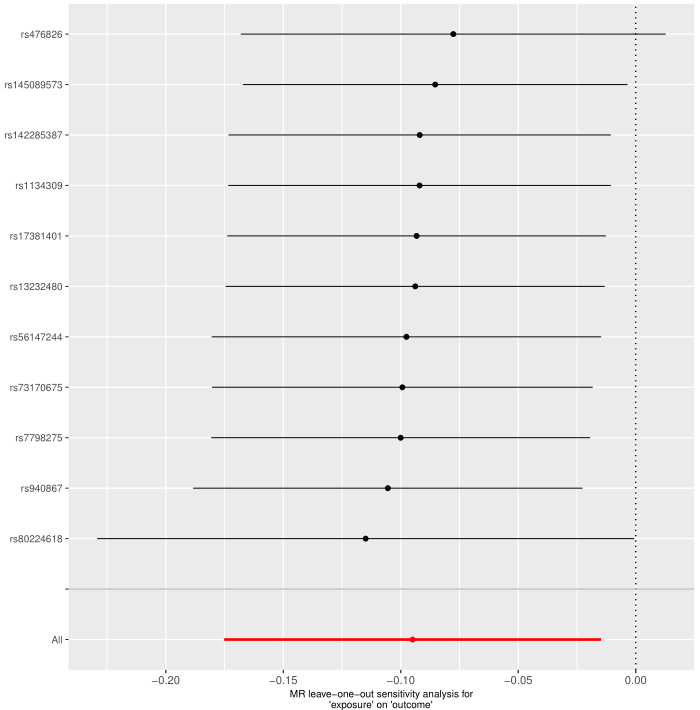


Figure S8. The eQTL data of GYPC gene expression and the result of MR analysis of AD.


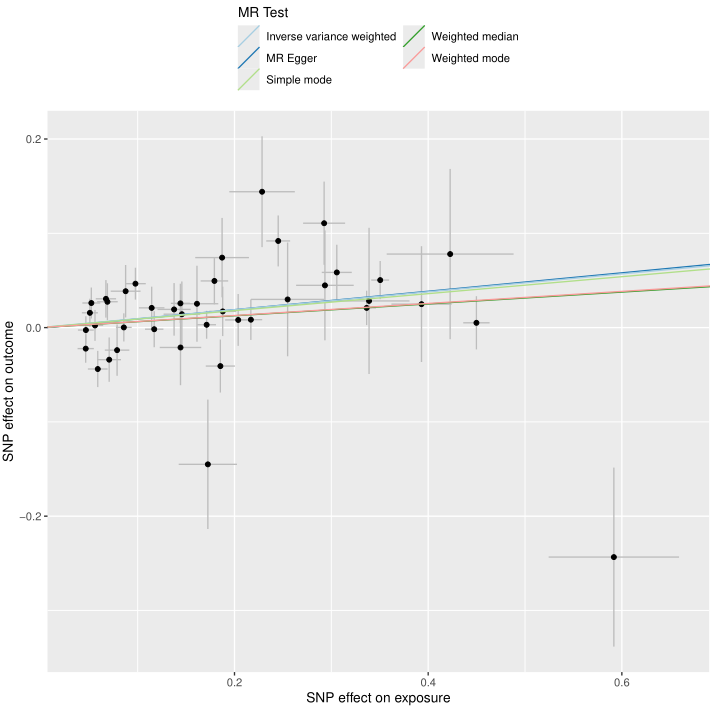


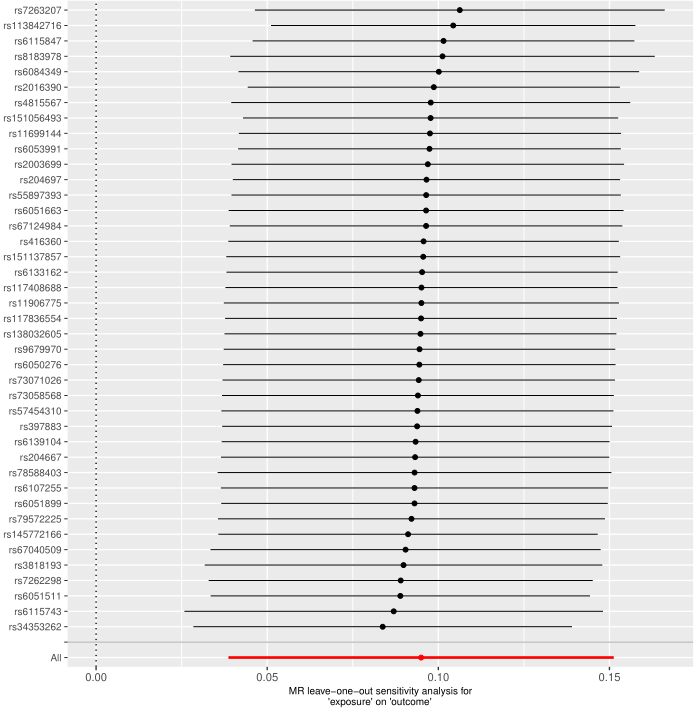


Figure S9. The eQTL data of TRIP11 gene expression and the result of MR analysis of AD.


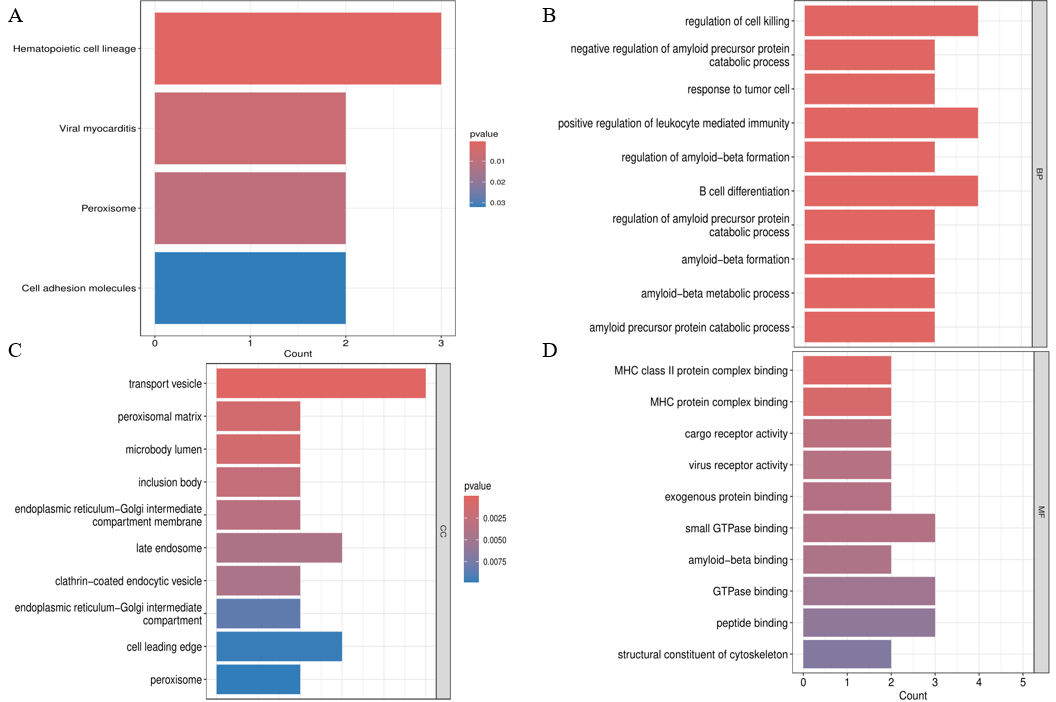


Figure S10. KEGG and GO analysis of AD GWAS variant sites in GTEx V8 blood tissues. (A) The KEGG pathway was used to analyse many key pathways, and different colours were used to represent related enriched genes. (B–D) Analysis of genes interacting with various biological processes, cellular components, and molecular functions.


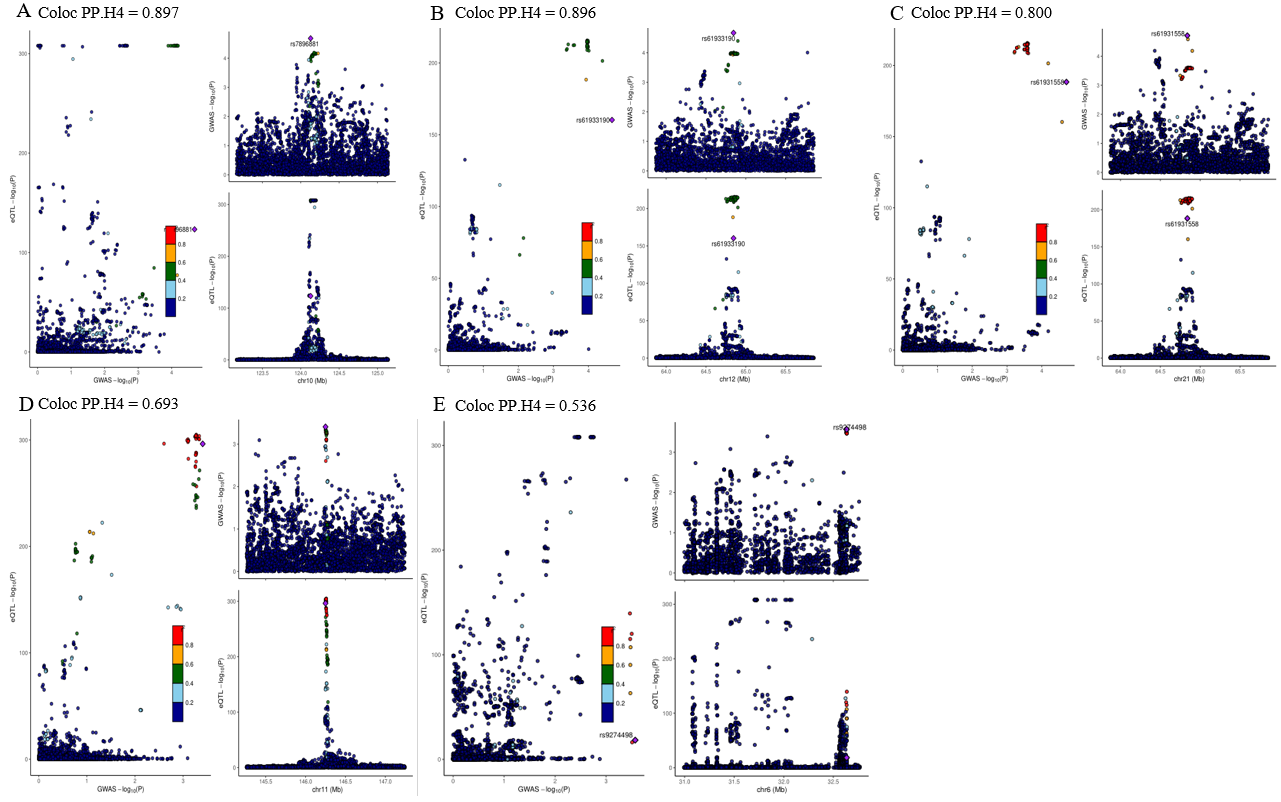


Figure S11.Colocalization analysis of cis-eQTLs and AD GWAS loci. Colocalized loci.PP.H4 > 0.5 between cis-eQTLs and AD GWAS signals. The r² values represent LD between each variant and the lead SNP. (A-E) Colocalization plots for PLEKHA1, TBK1, APP, PTGDR2, and CD28.


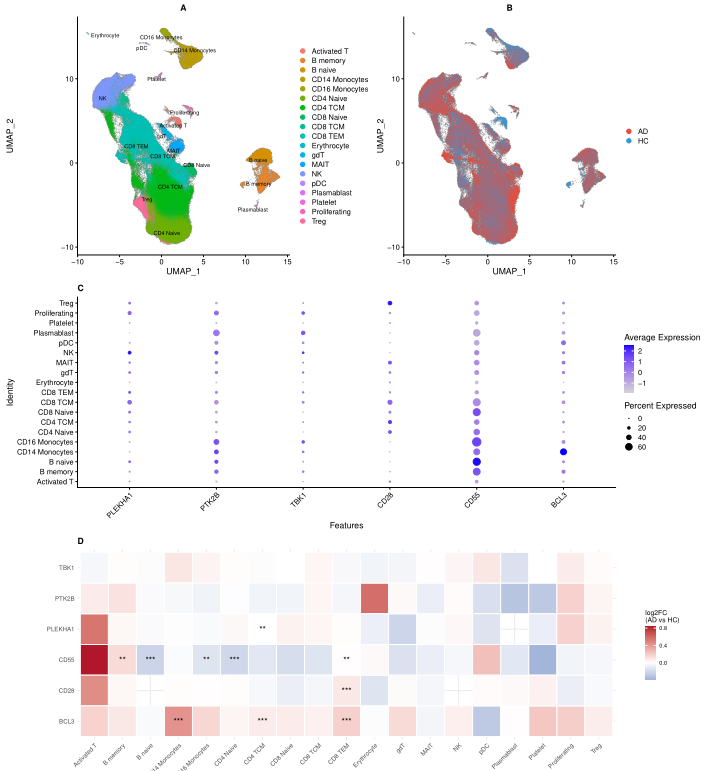


Figure S12. Independent validation of PBMC single-cell transcriptomics and candidate gene enrichment in the GSE226602 cohort. (A) UMAP visualization of 270,877 PBMCs from the validation cohort, annotated into 19 distinct cell types consistent with the original study nomenclature. (B) UMAP embedding colored by clinical diagnosis. (C) Dot plot illustrating the expression profiles of the six prioritized core candidate genes across all identified cell types. The dot size represents the percentage of cells expressing the gene, and the color intensity indicates the average expression level. (D) Differential expression analysis of the candidate genes between AD and HC within individual cell types. Significant gene-cell type associations (adjusted P < 0.05) are highlighted, demonstrating robust transcriptional alterations in the peripheral immune compartment.


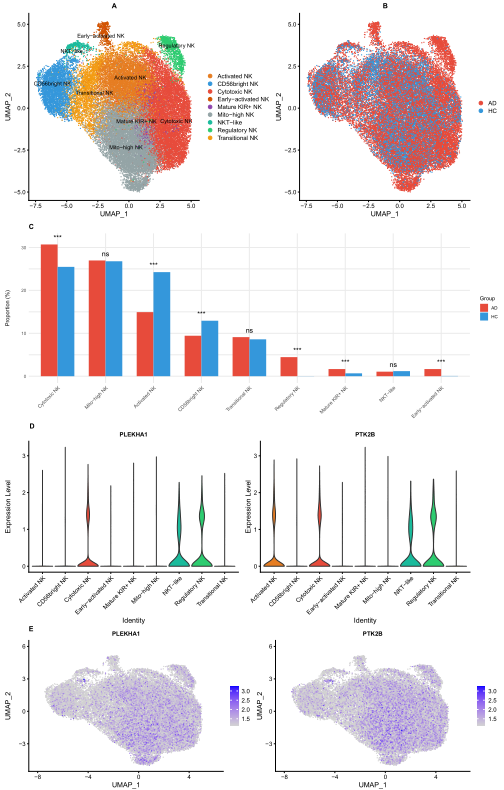


Figure S13. High-resolution sub-clustering reveals dramatic compositional remodeling of NK cells in Alzheimer's disease. (A) UMAP visualization of the NK cell compartment re-clustered into nine functional subtypes. (B) UMAP of NK cell subtypes stratified by clinical diagnosis. (C) Bar plot displaying the relative proportions of each NK cell subtype within the AD and HC groups. (D, E) Violin plots comparing the expression levels of targeted genes PLEKHA1 and PTK2B between AD and HC across the NK subtypes. "ns" indicates no statistical significance.


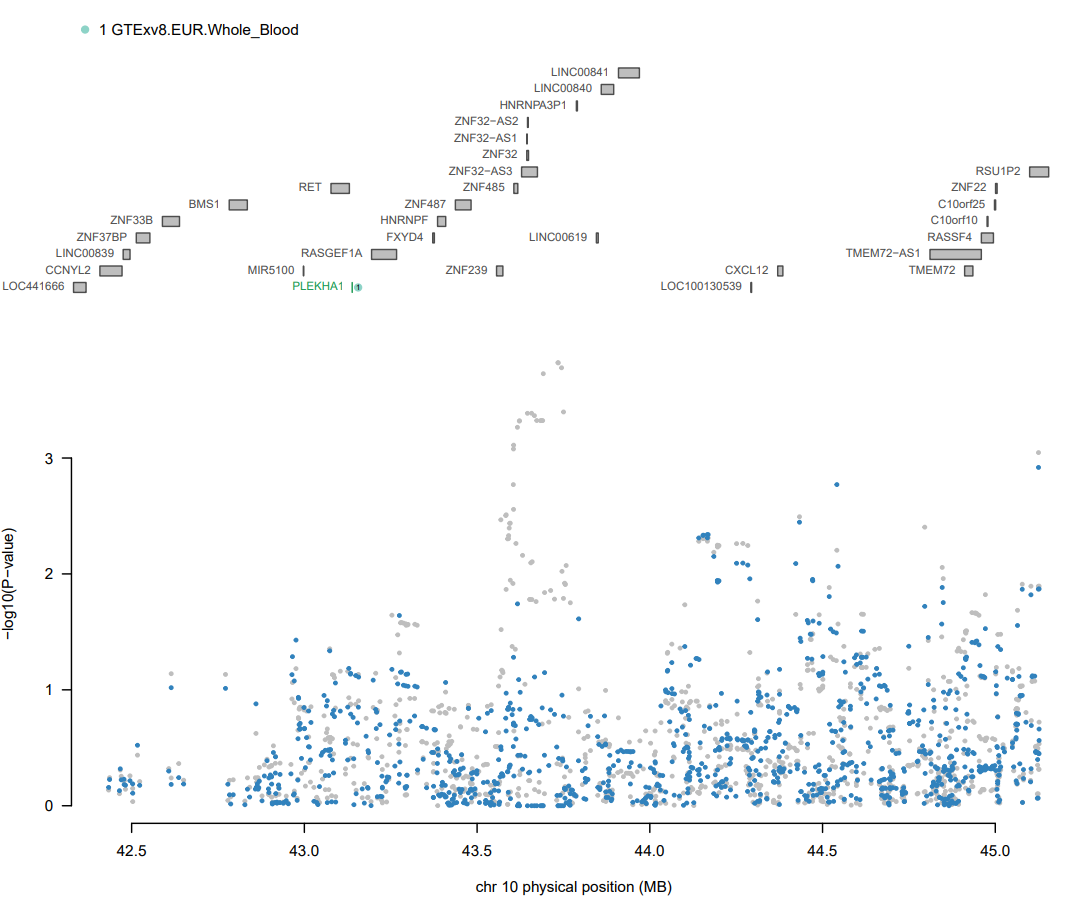


Figure S14. Regional association of TWAS hits for *PLEKHA1*. The top panel highlights all genes in the region. Marginally associated TWAS genes are shown in blue, and jointly significant genes are displayed in green. The bottom panel presents a regional Manhattan plot of GWAS data before (grey) and after (blue) conditioning on the predicted expression of the green-highlighted genes.
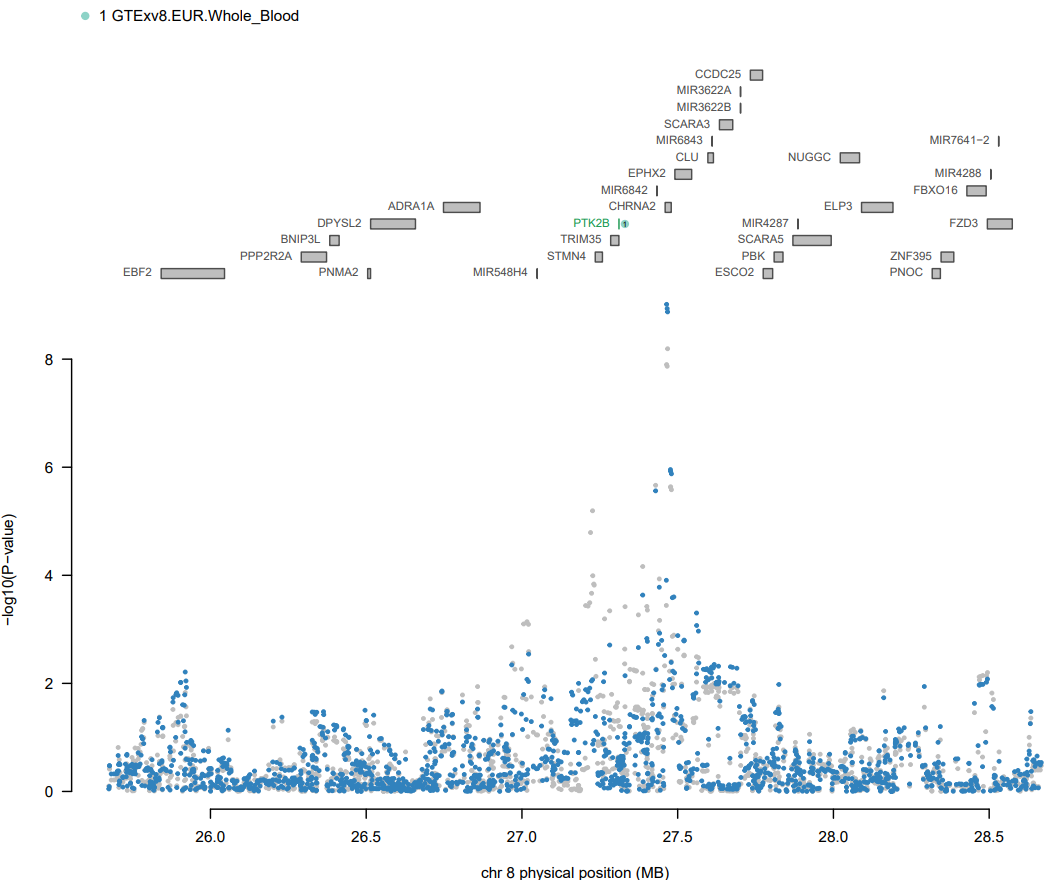


Figure S15. Regional association of TWAS hits for PTK2B. The top panel highlights all genes in the region. Marginally associated TWAS genes are shown in blue, and jointly significant genes are displayed in green. The bottom panel presents a regional Manhattan plot of GWAS data before (grey) and after (blue) conditioning on the predicted expression of the green-highlighted genes.


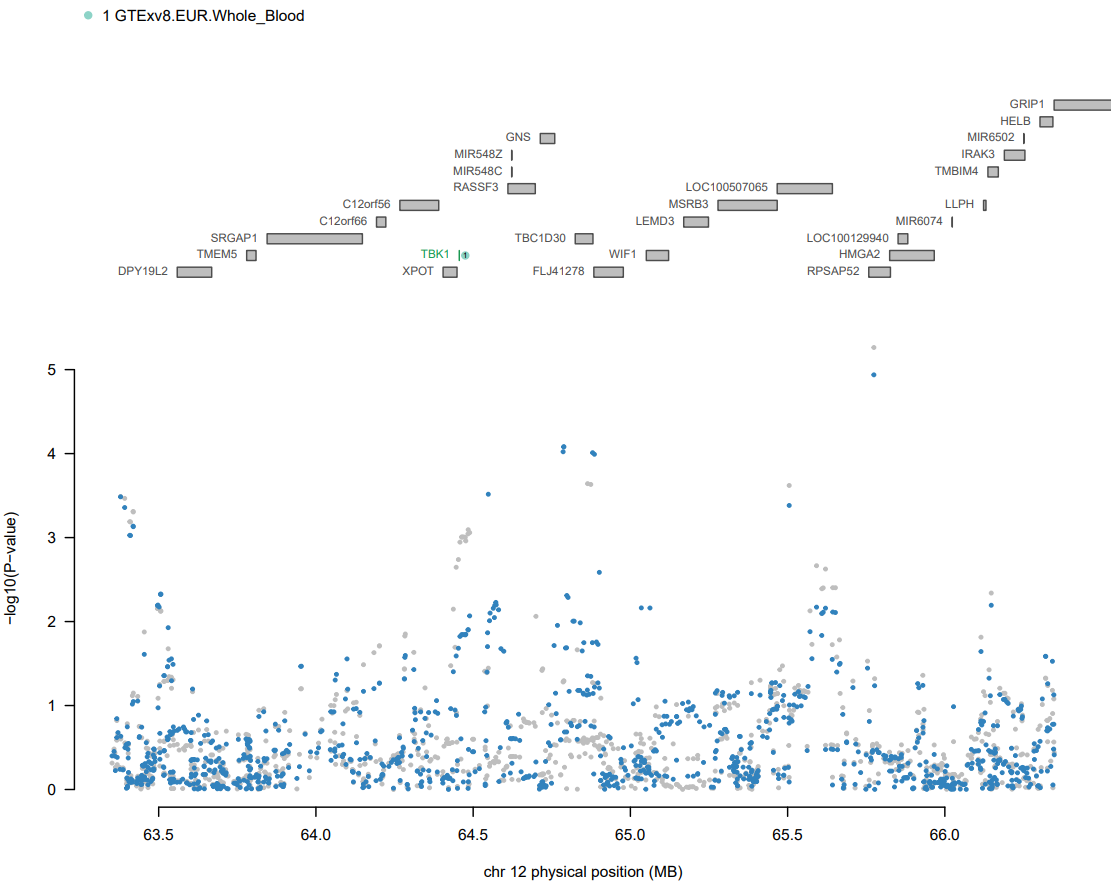


Figure S16. Regional association of TWAS hits for TBK1. The top panel highlights all genes in the region. Marginally associated TWAS genes are shown in blue, and jointly significant genes are displayed in green. The bottom panel presents a regional Manhattan plot of GWAS data before (grey) and after (blue) conditioning on the predicted expression of the green-highlighted genes.


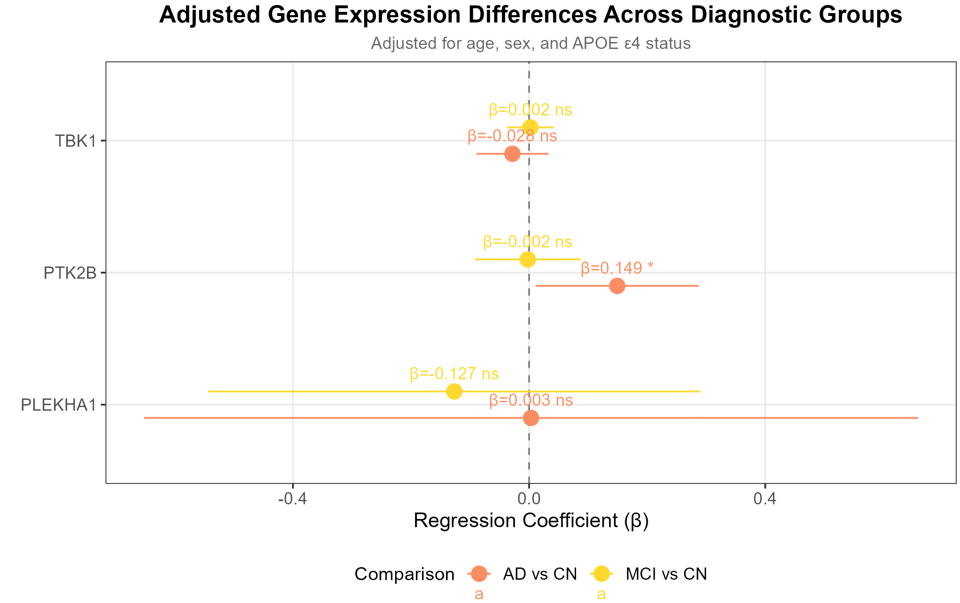


Figure S17.Covariate-adjusted regression of gene expression across diagnostic groups in the ADNI cohort. Forest plot showing regression coefficients (β) and 95% confidence intervals for diagnostic status (AD vs CN, MCI vs CN) associations with PLEKHA1, PTK2B, and TBK1 expression, adjusted for age, sex, and APOE ε4 status. Red and yellow circles denote β estimates for AD vs CN and MCI vs CN, respectively; P < 0.05; ns, not significant.


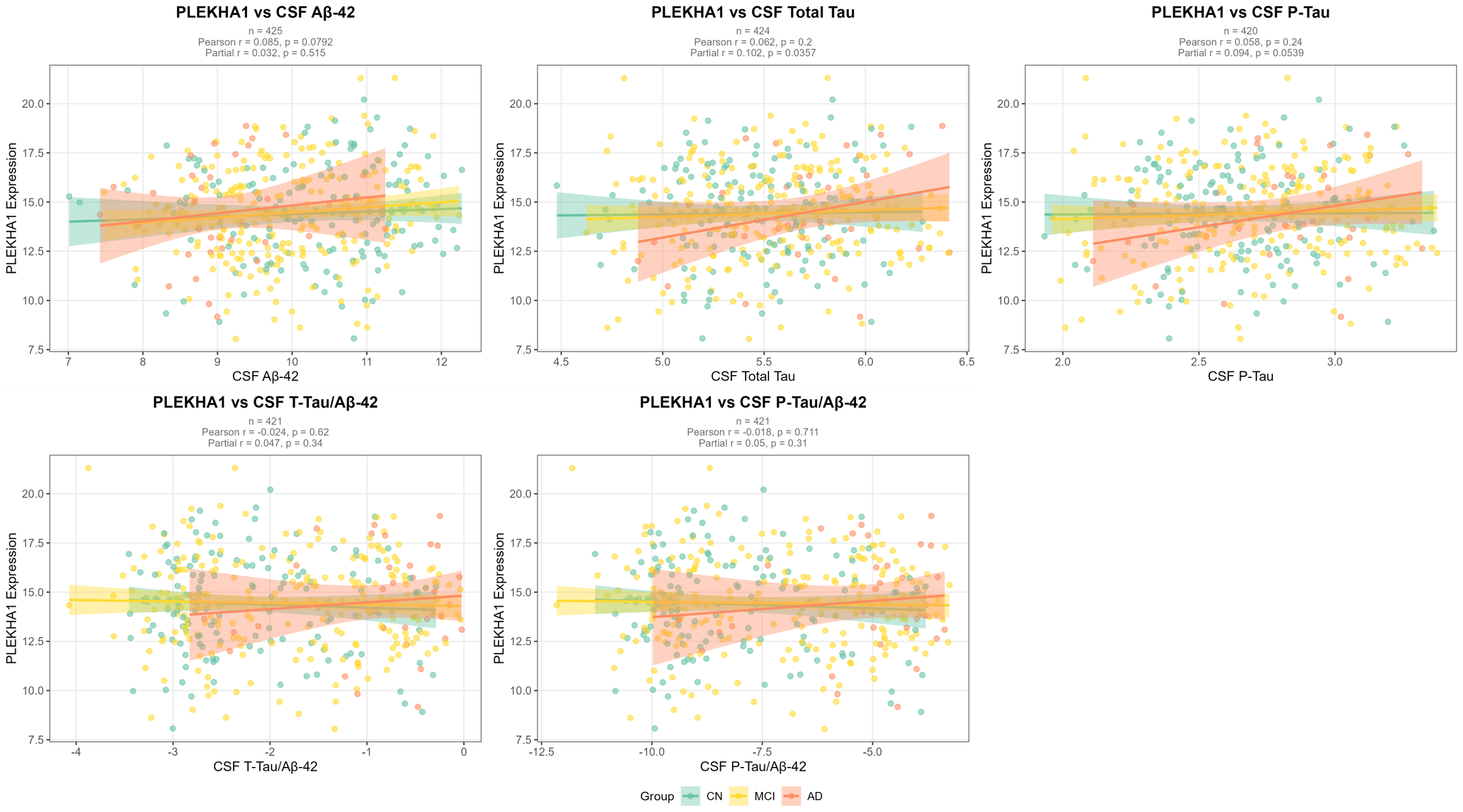


Figure S18. Associations between PLEKHA1 expression and CSF biomarkers in the ADNI cohort (N = 425). Scatter plots show Box-Cox-transformed PLEKHA1 expression versus transformed CSF Aβ42, total tau, phosphorylated tau, t-Tau/Aβ42 ratio, and p-Tau/Aβ42 ratio. Points are colored by diagnostic group (CN, green; MCI, yellow; AD, orange). Shaded bands indicate 95% confidence intervals for group-specific linear fits. Pearson correlations and partial correlations (adjusted for age, sex, and APOE ε4 status) are shown in each panel.


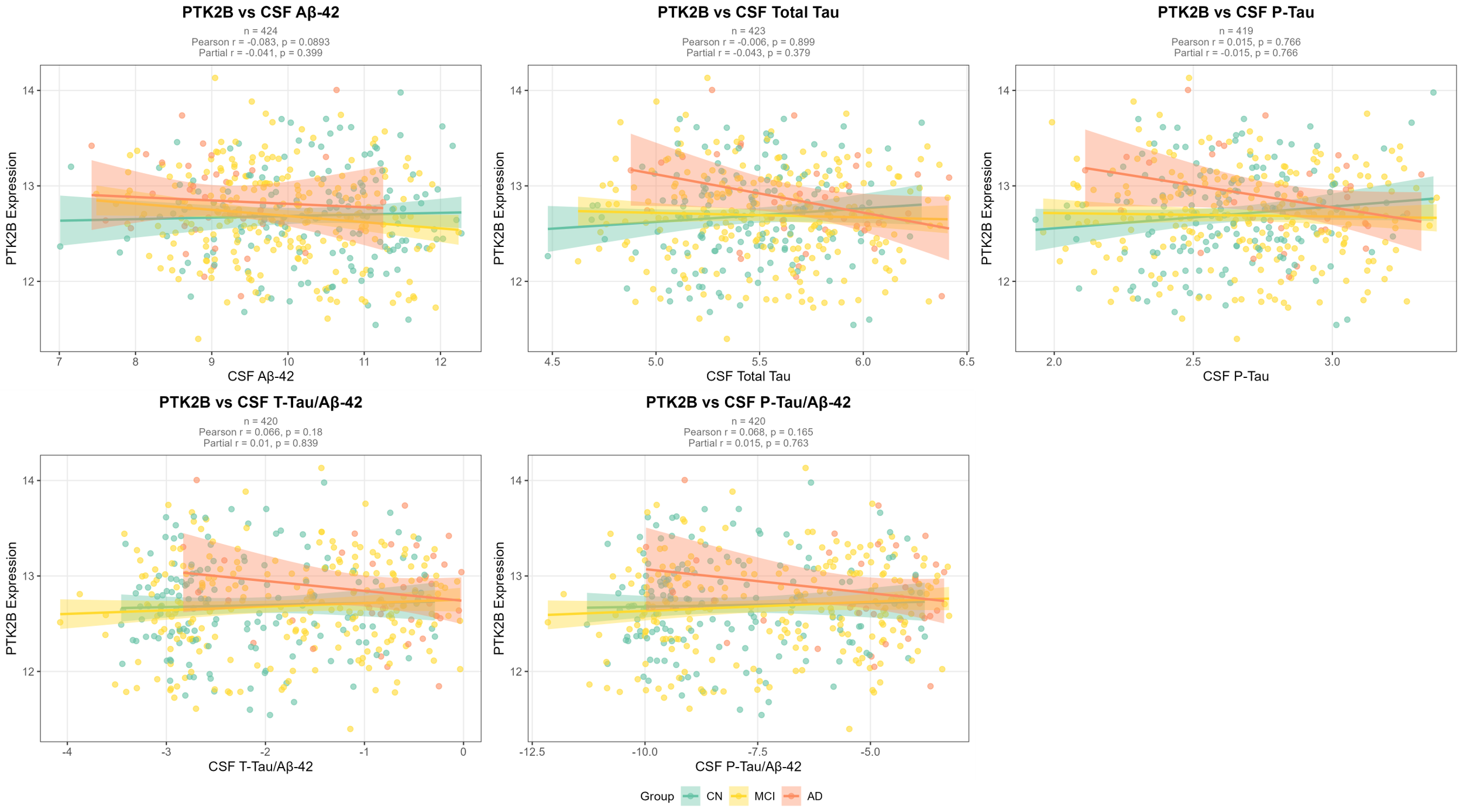


Figure S19. Associations between PTK2B expression and CSF biomarkers in the ADNI cohort (N = 425). Scatter plots show Box-Cox-transformed PTK2B expression versus transformed CSF Aβ42, total tau, phosphorylated tau (p-tau), t-Tau/Aβ42 ratio, and p-Tau/Aβ42 ratio. Points are colored by diagnostic group (CN, green; MCI, yellow; AD, orange). Shaded bands indicate 95% confidence intervals for group-specific linear fits. Pearson correlations and partial correlations (adjusted for age, sex, and APOE ε4 status) are shown in each panel.


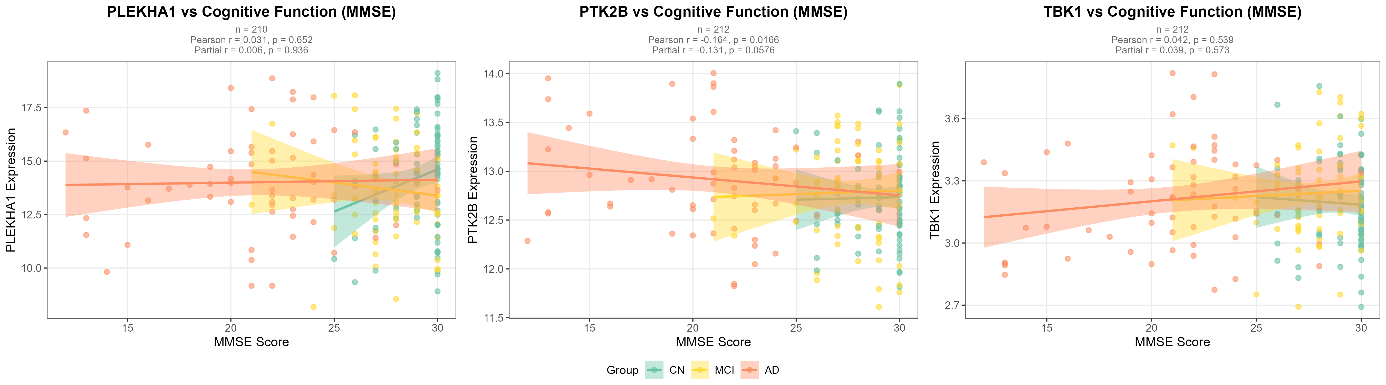


Figure S20. Associations between PLEKHA1, PTK2B, and TBK1 expression and cognitive function (MMSE) in the ADNI cohort (N = 425). Scatter plots show Box-Cox-transformed gene expression versus MMSE scores, with points colored by diagnostic group (CN, green; MCI, yellow; AD, orange). Shaded bands indicate 95% confidence intervals for group-specific linear fits. Pearson correlations and partial correlations (adjusted for age, sex, and APOE ε4 status) are annotated in each panel.


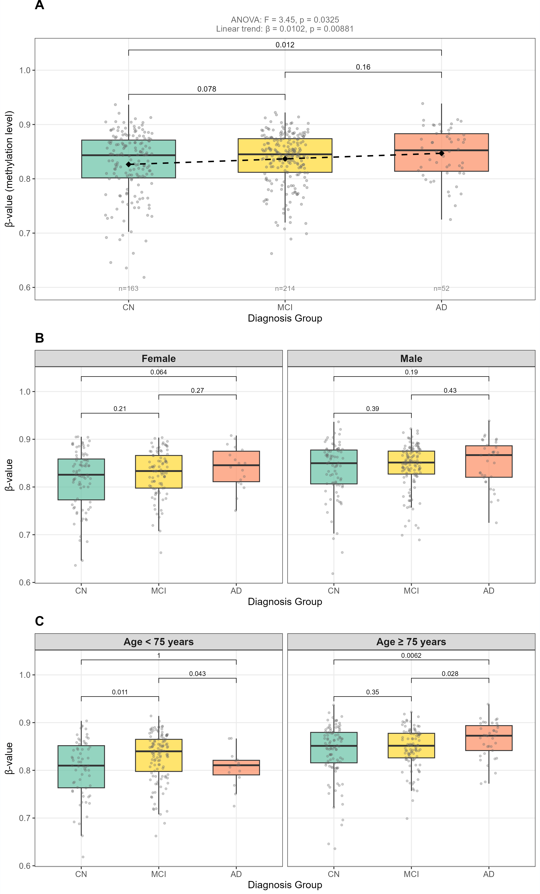


Figure S21.Subgroup analyses of cg19863426 methylation β-values across the cognitively normal (CN), mild cognitive impairment (MCI), and Alzheimer’s disease (AD) diagnostic spectrum. (A) Total cohort analysis of cg19863426 methylation β-values across CN, MCI, and AD diagnostic groups (ANOVA F = 3.45, P = 0.0325; linear trend β = 0.0102, P = 0.00881). (B) Sex-stratified subgroup analysis of cg19863426 methylation β-values across diagnostic groups in female and male participants. (C) Age-stratified subgroup analysis of cg19863426 methylation β-values across diagnostic groups in participants aged < 75 years and ≥ 75 years.


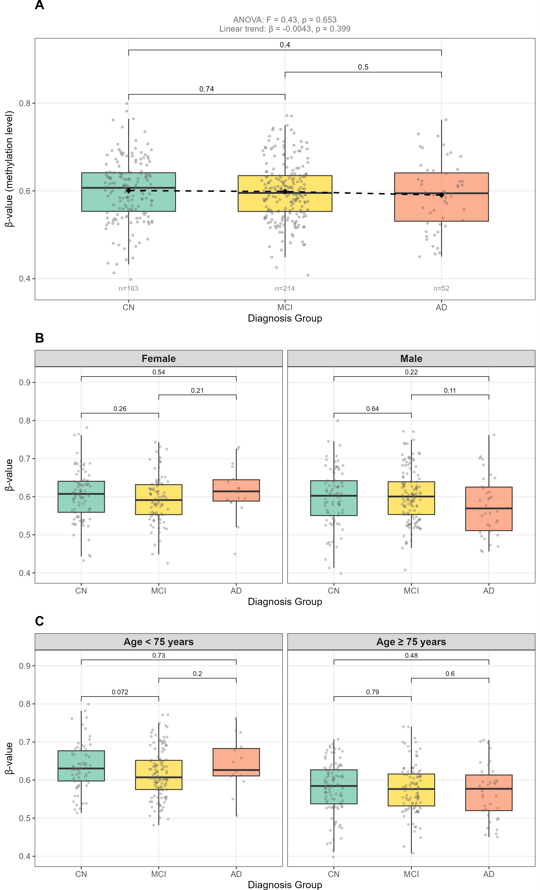


Figure S22.Subgroup analyses of cg16604658 methylation β-values across the cognitively normal (CN), mild cognitive impairment (MCI), and Alzheimer’s disease (AD) diagnostic spectrum. (A) Total cohort analysis of cg16604658 methylation β-values across CN, MCI, and AD diagnostic groups (ANOVA F = 0.43, P = 0.653; linear trend β = −0.0043, P = 0.399). (B) Sex-stratified subgroup analysis of cg16604658 methylation β-values across diagnostic groups in female and male participants. (C) Age-stratified subgroup analysis of cg16604658 methylation β-values across diagnostic groups in participants aged < 75 years and ≥ 75 years.


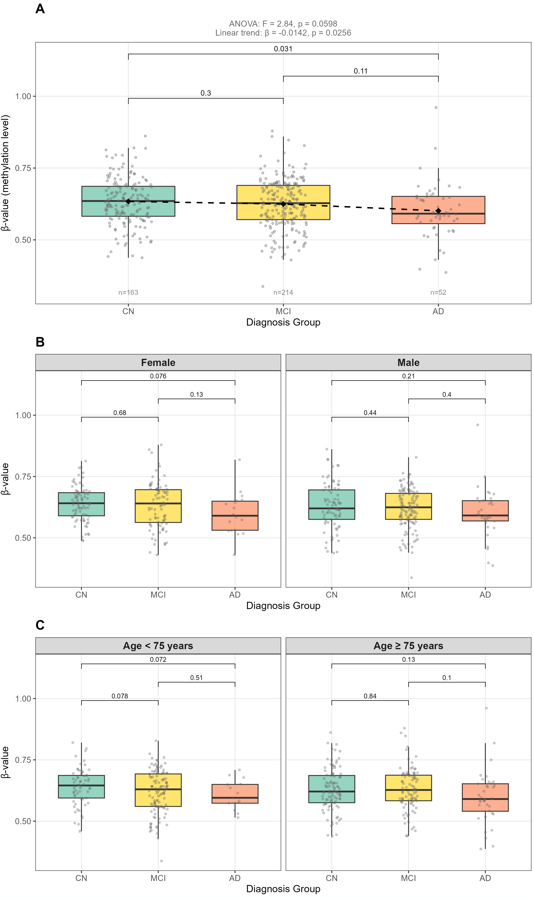


Figure S23. Subgroup analyses of cg14130459 (PTGDR2) methylation β-values across the cognitively normal (CN), mild cognitive impairment (MCI), and Alzheimer’s disease (AD) diagnostic spectrum. (A) Total cohort analysis of cg14130459 (PTGDR2) methylation β-values across CN, MCI, and AD diagnostic groups (ANOVA F = 2.84, P = 0.0598; linear trend β = −0.0142, P = 0.0256; post-hoc t-test AD vs CN P = 0.031). (B) Sex-stratified subgroup analysis of cg14130459 (PTGDR2) methylation β-values across diagnostic groups in female and male participants. (C) Age-stratified subgroup analysis of cg14130459 (PTGDR2) methylation β-values across diagnostic groups in participants aged < 75 years and ≥ 75 years.


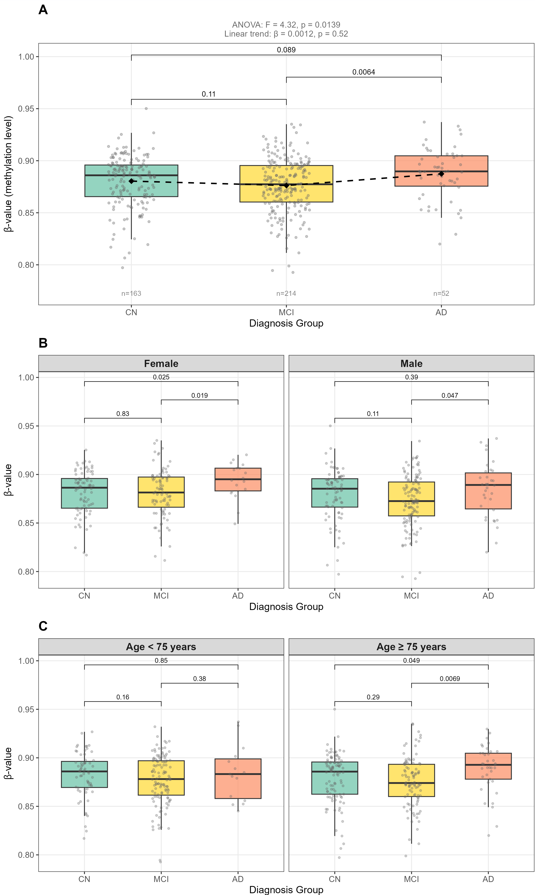


Figure S24. Subgroup analyses of cg19788250 (APP) methylation β-values across the cognitively normal (CN), mild cognitive impairment (MCI), and Alzheimer’s disease (AD) diagnostic spectrum. (A) Total cohort analysis of cg19788250 (APP) methylation β-values across CN, MCI, and AD diagnostic groups (ANOVA F = 4.32, P = 0.0139; linear trend β = 0.0152, P = 0.52; Tukey post-hoc MCI vs AD P = 0.013). (B) Sex-stratified subgroup analysis of cg19788250 (APP) methylation β-values across diagnostic groups in female and male participants. (C) Age-stratified subgroup analysis of cg19788250 (APP) methylation β-values across diagnostic groups in participants aged < 75 years and ≥ 75 years.


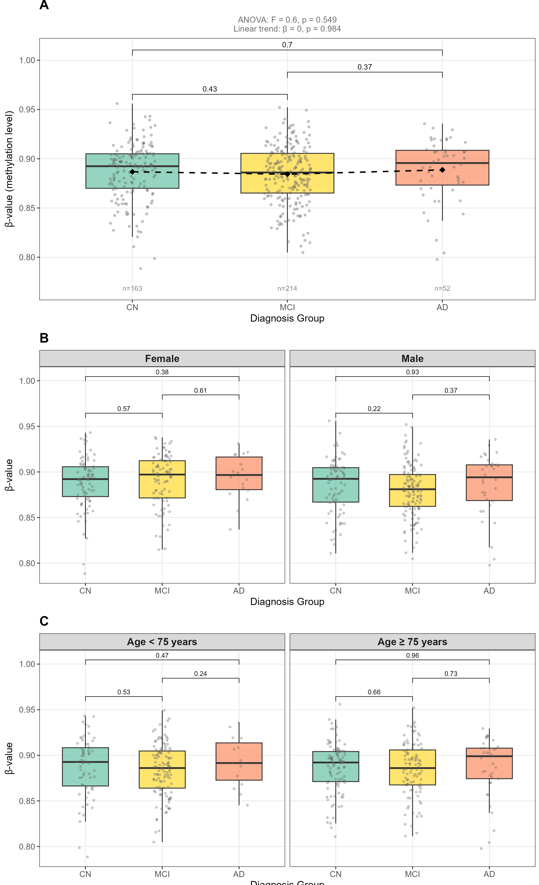


Figure S25.Subgroup analyses of cg01286133 (APP) methylation β-values across the cognitively normal (CN), mild cognitive impairment (MCI), and Alzheimer’s disease (AD) diagnostic spectrum. (A) Total cohort analysis of cg01286133 (APP) methylation β-values across CN, MCI, and AD diagnostic groups (ANOVA F = 0.6, P = 0.549; linear trend β = 0, P = 0.984). (B) Sex-stratified subgroup analysis of cg01286133 (APP) methylation β-values across diagnostic groups in female and male participants. (C) Age-stratified subgroup analysis of cg01286133 (APP) methylation β-values across diagnostic groups in participants aged < 75 years and ≥ 75 years.


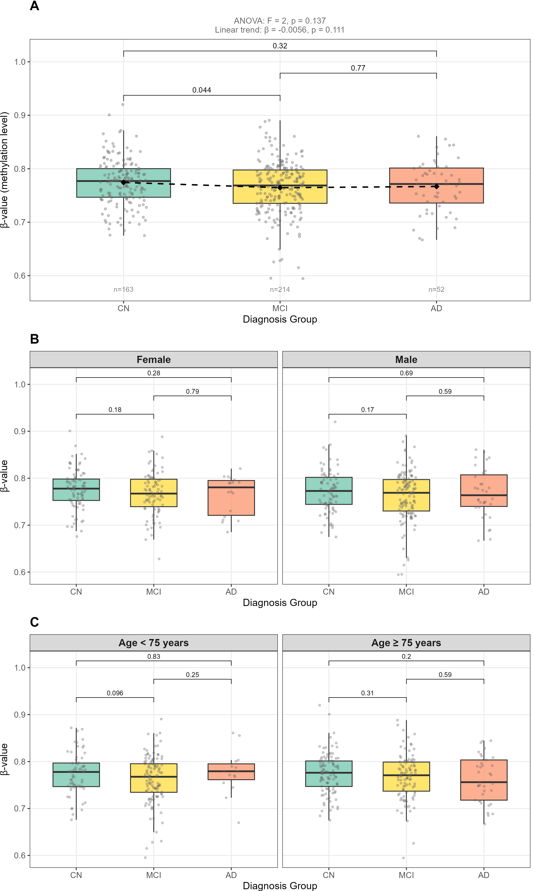


Figure S26.Subgroup analyses of cg18597421 (APP) methylation β-values across the cognitively normal (CN), mild cognitive impairment (MCI), and Alzheimer’s disease (AD) diagnostic spectrum. (A) Total cohort analysis of cg18597421 (APP) methylation β-values across CN, MCI, and AD diagnostic groups (ANOVA F = 2, P = 0.137; linear trend β = −0.0056, P = 0.111). (B) Sex-stratified subgroup analysis of cg18597421 (APP) methylation β-values across diagnostic groups in female and male participants. (C) Age-stratified subgroup analysis of cg18597421 (APP) methylation β-values across diagnostic groups in participants aged < 75 years and ≥ 75 years.


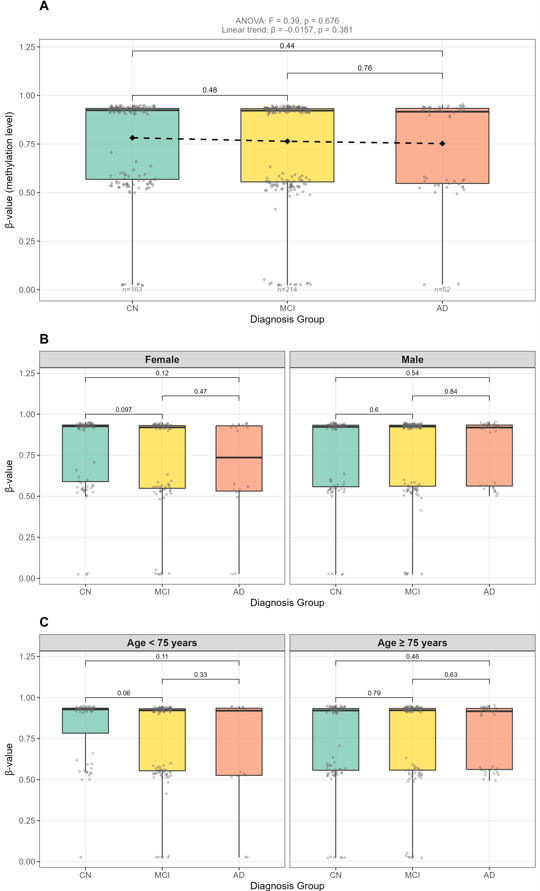


Figure S27.Subgroup analyses of cg05092371 (CD28) methylation β-values across the cognitively normal (CN), mild cognitive impairment (MCI), and Alzheimer’s disease (AD) diagnostic spectrum. (A) Total cohort analysis of cg05092371 (CD28) methylation β-values across CN, MCI, and AD diagnostic groups (ANOVA F = 0.39, P = 0.676; linear trend β = −0.0157, P = 0.381). (B) Sex-stratified subgroup analysis of cg05092371 (CD28) methylation β-values across diagnostic groups in female and male participants. (C) Age-stratified subgroup analysis of cg05092371 (CD28) methylation β-values across diagnostic groups in participants aged < 75 years and ≥ 75 years.


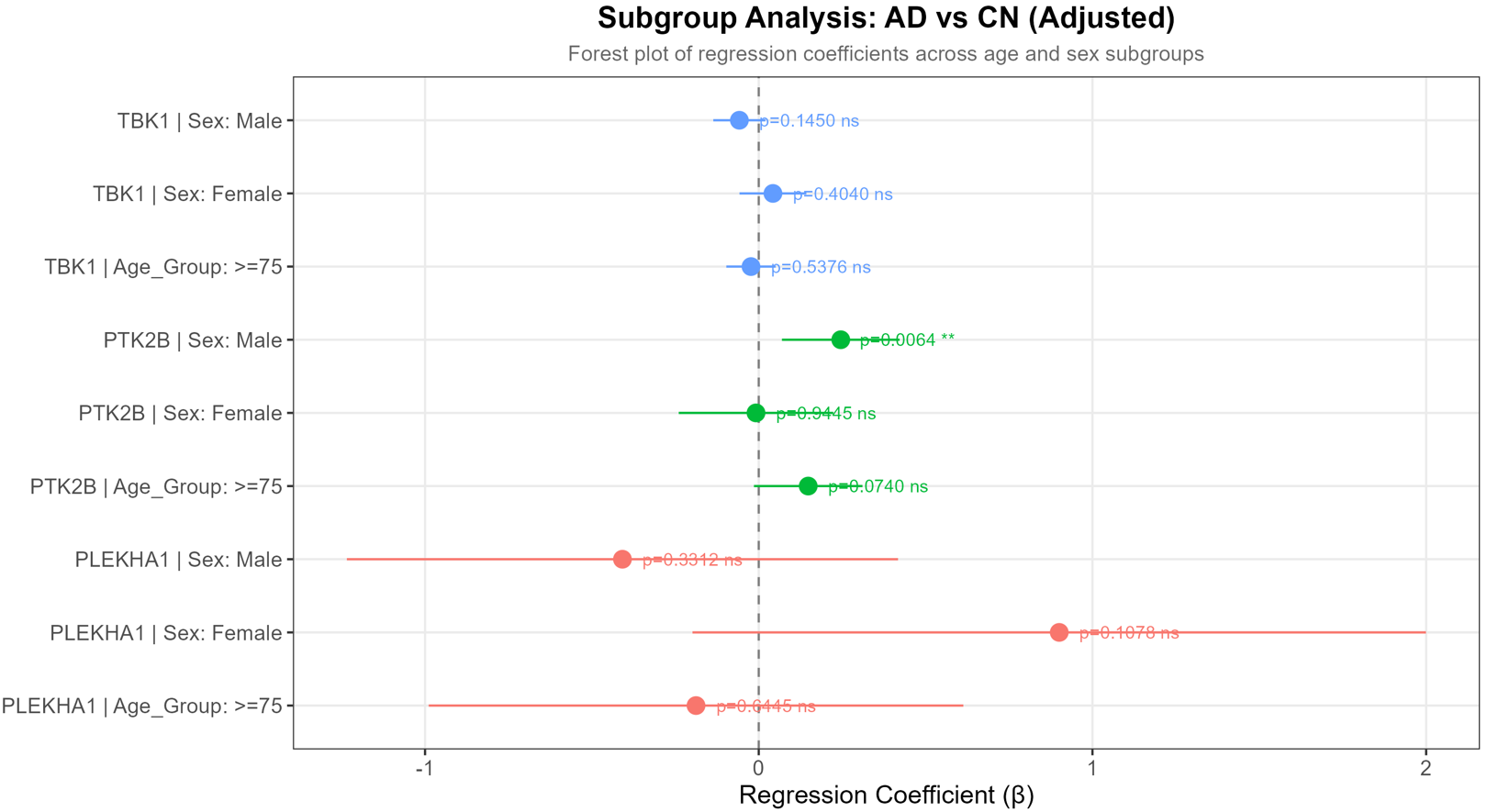


Figure S28. Subgroup analysis of AD-associated gene methylation effects on AD risk (AD vs CN, adjusted).
